# Gender Difference in the Effects of COVID-19 Pandemic on Mechanical Reperfusion and 30-Day Mortality for STEMI: Results of the ISACS-STEMI COVID-19 Registry

**DOI:** 10.3390/jcm12030896

**Published:** 2023-01-23

**Authors:** Giuseppe De Luca, Stephane Manzo-Silberman, Magdy Algowhary, Berat Uguz, Dinaldo C. Oliveira, Vladimir Ganyukov, Oliver Busljetik, Miha Cercek, Lisette Okkels, Poay Huan Loh, Lucian Calmac, Gerard Roura i Ferrer, Alexandre Quadros, Marek Milewski, Fortunato Scotto di Uccio, Clemens von Birgelen, Francesco Versaci, Jurrien Ten Berg, Gianni Casella, Aaron Wong Sung Lung, Petr Kala, José Luis Díez Gil, Xavier Carrillo, Maurits Dirksen, Victor Becerra, Michael Kang-yin Lee, Dafsah Arifa Juzar, Rodrigo de Moura Joaquim, Roberto Paladino, Davor Milicic, Periklis Davlouros, Nikola Bakraceski, Filippo Zilio, Luca Donazzan, Adriaan Kraaijeveld, Gennaro Galasso, Lux Arpad, Lucia Marinucci, Vincenzo Guiducci, Maurizio Menichelli, Alessandra Scoccia, Aylin Hatice Yamac, Kadir Ugur Mert, Xacobe Flores Rios, Tomas Kovarnik, Michal Kidawa, Josè Moreu, Vincent Flavien, Enrico Fabris, Iñigo Lozano Martínez-Luengas, Marco Boccalatte, Francisco Bosa Ojeda, Carlos Arellano-Serrano, Gianluca Caiazzo, Giuseppe Cirrincione, Hsien-Li Kao, Juan Sanchis Forés, Luigi Vignali, Helder Pereira, Santiago Ordoñez, Alev Arat Özkan, Bruno Scheller, Heidi Lehtola, Rui Teles, Christos Mantis, Ylitalo Antti, João António Brum Silveira, Cesar Rodrigo Zoni, Ivan Bessonov, Giuseppe Uccello, George Kochiadakis, Dimitrios Alexopulos, Carlos E. Uribe, John Kanakakis, Benjamin Faurie, Gabriele Gabrielli, Alejandro Gutierrez Barrios, Juan Pablo Bachini, Alex Rocha, Frankie C. C. Tam, Alfredo Rodriguez, Antonia Anna Lukito, Veauthyelau Saint-Joy, Gustavo Pessah, Andrea Tuccillo, Alfonso Ielasi, Giuliana Cortese, Guido Parodi, Mohamed Abed Bouraghda, Marcia Moura, Elvin Kedhi, Pablo Lamelas, Harry Suryapranata, Matteo Nardin, Monica Verdoia

**Affiliations:** 1 Division of Cardiology, AOU “Policlinico G. Martino”, Department of Clinical and Experimental Medicine, University of Messina, 98124 Messina, Italy; 2 Division of Cardiology, Nuovo Galeazzi-Sant’Ambrogio Hospital, 20161 Milan, Italy; 3 ACTION Study Group, Institute of Cardiology—Hôpital Pitié-Salpêtrière (AP-HP), Sorbonne University, 75651 Paris, France; 4 Division of Cardiology, Assiut University Heart Hospital, Assiut University, Asyut 71515, Egypt; 5 Division of Cardiology, Bursa City Hospital, 16000 Bursa, Turkey; 6 Pronto de Socorro Cardiologico Prof. Luis Tavares, Centro PROCAPE, Federal University of Pernambuco, Recife 50670-901, Brazil; 7 Department of Heart and Vascular Surgery, State Research Institute for Complex Issues of Cardiovascular Diseases, 650000 Kemerovo, Russia; 8 University Clinic for Cardiology, Medical Faculty, Ss’ Cyril and Methodius University, 1000 Skopje, North Macedonia; 9 Department of Cardiology, Medical Center Ljubljana, 1000 Ljubljana, Slovenia; 10 Division of Cardiology, Odense Universitets Hospital, 5000 Odense, Denmark; 11 Department of Cardiology, National University Hospital, Singapore 117597, Singapore; 12 Clinic Emergency Hospital of Bucharest, 014461 Bucharest, Romania; 13 Interventional Cardiology Unit, Heart Disease Institute, Hospital Universitari de Bellvitge, 08016 Barcelona, Spain; 14 Instituto de Cardiologia do Rio Grande do Sul, Porto Alegre 90040-371, Brazil; 15 Division of Cardiology, Medical University of Silezia, 40-002 Katowice, Poland; 16 Division of Cardiology, Ospedale del Mare, 00156 Napoli, Italy; 17 Department of Cardiology, Medisch Spectrum Twente, Thoraxcentrum Twente, 7541 Enschede, The Netherlands; 18 Division of Cardiology, Ospedale Santa Maria Goretti Latina, 04100 Latina, Italy; 19 Division of Cardiology, St. Antonius Hospital, 3434 Nieuwegein, The Netherlands; 20 Division of Cardiology, Ospedale Maggiore Bologna, 40100 Bologna, Italy; 21 Department of Cardiology, National Heart Center, Singapore 169609, Singapore; 22 University Hospital Brno, Medical Faculty of Masaryk University, 60200 Brno, Czech Republic; 23 Sección de la Unidad de Hemodinámica H. Universitario y Politécnico La Fe, 46001 Valencia, Spain; 24 Hospital Germans Triasi Pujol, 8918 Badalona, Spain; 25 Division of Cardiology, Northwest Clinics, 1811 Alkmaar, The Netherlands; 26 Hospital Clínico Universitario Virgen de la Victoria, 29000 Malaga, Spain; 27 Department of Cardiology, Queen Elizabeth Hospital, University of Hong Kong, Hong Kong; 28 Department of Cardiology and Vascular Medicine, University of Indonesia National Cardiovascular Center “Harapan Kita”, Jakarta 11420, Indonesia; 29 Instituto de Cardiologia de Santa Catarina Praia Comprida, São José 88103-901, Brazil; 30 Division of Cardiology, Clinica Villa dei Fiori, 80011 Acerra, Italy; 31 Department of Cardiology, University Hospital Centre, University of Zagreb, 10000 Zagreb, Croatia; 32 Invasive Cardiology and Congenital Heart Disease, Patras University Hospital, 26221 Patras, Greece; 33 Center for Cardiovascular Diseases, 6000 Ohrid, North Macedonia; 34 Division of Cardiology, Ospedale Santa Chiara di Trento, 38014 Trento, Italy; 35 Division of Cardiology, Ospedale “S. Maurizio” Bolzano, 39100 Bolzano, Italy; 36 Division of Cardiology, 3584 Utrecht, The Netherlands; 37 Division of Cardiology, Ospedale San Giovanni di Dio e Ruggi d’Aragona, 84070 Salerno, Italy; 38 Maastricht University Medical Center, 6229 Maastricht, The Netherlands; 39 Division of Cardiology, Azienda Ospedaliera “Ospedali Riuniti Marche Nord”, 61121 Pesaro, Italy; 40 Division of Cardiology, AUSL-IRCCS Reggio Emilia, 42121 Reggio Emilia, Italy; 41 Division of Cardiology, Ospedale “F. Spaziani”, 03100 Frosinone, Italy; 42 Division of Cardiology, Ospedale “Sant’Anna”, 44121 Ferrara, Italy; 43 Department of Cardiology, Bezmialem Vakif University Hospital, 34093 Istanbul, Turkey; 44 Division of Cardiology, Faculty of Medicine, Eskisehir Osmangazi University, 02640 Eskisehir, Turkey; 45 Complexo Hospetaliero Universitario La Coruna, 15001 La Coruna, Spain; 46 University Hospital Prague, 12808 Prague, Czech Republic; 47 Central Hospital of Medical University of Lodz, 90-008 Lodz, Poland; 48 Division of Cardiology, Complejo Hospitalario de Toledo, 45001 Toledo, Spain; 49 Division of Cardiology, Center Hospitalier Universitaire de Lille, 59000 Lille, France; 50 Azienda Ospedaliero—Universitaria Ospedali Riuniti, 34142 Trieste, Italy; 51 Division of Cardiology, Hospital Cabueñes, 33201 Gijon, Spain; 52 Division of Cardiology, Ospedale Santa Maria delle Grazie, 80078 Pozzuoli, Italy; 53 Division of Cardiology, Hospital Universitario de Canarias, 38001 Santa Cruz de Tenerife, Spain; 54 Division of Cardiology, Hospital Puerta de Hierro Majadahonda, 28222 Majadahonda, Spain; 55 Division of Cardiology, Ospedale “G Moscati”, 81031 Aversa, Italy; 56 Division of Cardiology, Ospedale Civico Arnas, 90100 Palermo, Italy; 57 Cardiology Division, Department of Internal Medicine, National Taiwan University Hospital, Tapei 8865600, Taiwan; 58 Division of Cardiology, Hospital Clinico Universitario de Valencia, 46010 Valencia, Spain; 59 Interventional Cardiology Unit, Azienda Ospedaliera Sanitaria, 43121 Parma, Italy; 60 Cardiology Department, Hospital Garcia de Orta, Pragal, 2805-267 Almada, Portugal; 61 Instituto Cardiovascular de Buenos Aires, Buenos Aires 1428, Argentina; 62 Cardiology Institute, Istanbul University, 34000 Instanbul, Turkey; 63 Division of Cardiology, Clinical and Experimental Interventional Cardiology, University of Saarland, 66123 Saarbrücken, Germany; 64 Division of Cardiology, Oulu University Hospital, 90220 Oulu, Finland; 65 Division of Cardiology, Hospital de Santa Cruz, CHLO—Nova Medical School, CEDOC, 1000 Lisbon, Portugal; 66 Division of Cardiology, Kontantopoulion Hospital, 10431 Athens, Greece; 67 Division of Cardiology, Heart Centre Turku, 20521 Turku, Finland; 68 Division of Cardiology, Hospital de Santo António, 4099-001 Porto, Portugal; 69 Department of Teaching and Research, Instituto de Cardiología de Corrientes “Juana F. Cabral”, Corrientes W3400CDS, Argentina; 70 Tyumen Cardiology Research Center, Tomsk National Research Medical Center, Russian Academy of Science, 634000-634538 Tomsk, Russia; 71 Division of Cardiology, Ospedale “A. Manzoni”, 23900 Lecco, Italy; 72 Iraklion University Hospital, 70001 Crete, Greece; 73 Division of Cardiology, Attikon University Hospital, 10431 Athens, Greece; 74 Division of Cardiology, Universidad UPB, Universidad CES, Medellín 050001, Colombia; 75 Division of Cardiology, Alexandra Hospital, 10431 Athens, Greece; 76 Division of Cardiology, Groupe Hospitalier Mutualiste de Grenoble, 38000 Grenoble, France; 77 Interventional Cardiology Unit, Azienda Ospedaliero Universitaria“Ospedali Riuniti”, 60100 Ancona, Italy; 78 Division of Cardiology, Hospital Puerta del Mar, 11001 Cadiz, Spain; 79 Instituto de Cardiologia Integral, Montevideo 11700, Uruguay; 80 Department of Cardiology and Cardiovascular Interventions, Instituto Nacional de Cirugía Cardíaca, Montevideo 11700, Uruguay; 81 Department of Cardiology, Queen Mary Hospital, University of Hong Kong, Hong Kong; 82 Division of Cardiology, Otamendi Hospital, Buenos Aires 1001, Argentina; 83 Cardiovascular Department Pelita Harapan University, Heart Center Siloam Lippo Village Hospital, Tangerang, Banten 15810, Indonesia; 84 Center Hospitalier d’Antibes Juan Les Pins, 06600 Antibes, France; 85 Division of Cardiology, Hospiatl Cordoba, Cordoba 5000, Argentina; 86 Department of Statistical Sciences, University of Padova, 35121 Padova, Italy; 87 Azienda Ospedaliera Lavagna, 16033 Lavagna, Italy; 88 Division of Cardiology, Blida University Hospital, Blida 09000, Algeria; 89 Division of Cardiology, Hopital Erasmus, Universitè Libre de Bruxelles, 1070 Brussels, Belgium; 90 Division of Cardiology, Radboud University Medical Center, 6525 Nijmegen, The Netherlands; 91 Department of Internal Medicine, Ospedale Riuniti, 25121 Brescia, Italy; 92 Division of Cardiology, Ospedale degli Infermi, ASL, 13900 Biella, Italy

**Keywords:** gender, ST-segment elevation myocardial infarction, percutaneous coronary intervention, COVID-19

## Abstract

Background. Several reports have demonstrated the impact of the COVID-19 pandemic on the management and outcome of patients with ST-segment elevation myocardial infarction (STEMI). The aim of the current analysis is to investigate the potential gender difference in the effects of the COVID-19 pandemic on mechanical reperfusion and 30-day mortality for STEMI patients within the ISACS-STEMI COVID-19 Registry. Methods. This retrospective multicenter registry was performed in high-volume primary percutaneous coronary intervention (PPCI) centers on four continents and included STEMI patients undergoing PPCIs in March–June 2019 and 2020. Patients were divided according to gender. The main outcomes were the incidence and timing of the PPCI, (ischemia time ≥ 12 h and door-to-balloon ≥ 30 min) and in-hospital or 30-day mortality. Results. We included 16683 STEMI patients undergoing PPCIs in 109 centers. In 2020 during the pandemic, there was a significant reduction in PPCIs compared to 2019 (IRR 0.843 (95% CI: 0.825–0.861, *p* < 0.0001). We did not find a significant gender difference in the effects of the COVID-19 pandemic on the numbers of STEMI patients, which were similarly reduced from 2019 to 2020 in both groups, or in the mortality rates. Compared to prepandemia, 30-day mortality was significantly higher during the pandemic period among female (12.1% vs. 8.7%; adjusted HR [95% CI] = 1.66 [1.31–2.11], *p* < 0.001) but not male patients (5.8% vs. 6.7%; adjusted HR [95% CI] = 1.14 [0.96–1.34], *p* = 0.12). Conclusions. The COVID-19 pandemic had a significant impact on the treatment of patients with STEMI, with a 16% reduction in PPCI procedures similarly observed in both genders. Furthermore, we observed significantly increased in-hospital and 30-day mortality rates during the pandemic only among females. *Trial registration number: NCT 04412655.*

## 1. Introduction

The SARS-CoV-2 virus has largely and rapidly spread across the world, infecting more than 300 million people, with more than 5 million deaths [1,2]. The COVID-19 pandemic has severely impacted our healthcare systems, with the subsequent suspension of elective procedures and treatment of chronic conditions. Despite the maintenance of services for the management of urgent conditions, such as acute coronary syndromes, several previous reports have clearly shown a reduction in the number of treated acute coronary cases, mainly due to the fear of contagion that prevents patient presentation to hospitals [3,4,5]. Additionally, the time from symptom onset to treatment has significantly risen [6,7,8], secondary to the oversaturation of emergency departments, which contributes to explaining the higher mortality among STEMI patients observed in 2020. These results were confirmed by the recent International Study on Acute Coronary Syndromes—ST Elevation Myocardial Infarction (ISACS-STEMI) COVID-19 Registry [9], which provided a snapshot on the treatment and outcomes of STEMI patients treated with primary angioplasty during the COVID-19 pandemic in more than one hundred hospitals worldwide.

Male gender has been associated with a higher risk of COVID-19 infection compared to females, with higher rates of complications and worse outcomes [10,11]. Oppositely, among STEMI patients, females generally display more comorbidities and higher mortality [12,13]. However, thus far no study has investigated any potential gender difference in the impact of COVID-19 on STEMI cases treated percutaneously or clinical outcomes, with special attention to major high-risk features such as age, diabetes, smoking, and hypertension [14], which is the aim of the current study.

## 2. Methods

### Study Design and Population

This is a large-scale retrospective multicenter registry promoted by Eastern Piedmont University, Novara, Italy. The initial planning included European primary PCI centers [9], but the study was subsequently extended to several other regions in different continents (Latin America, southeast Asia, and north Africa). Included centers were required to perform more than 120 primary PCI/year (with an expected average >10/month), with the STEMI caseload not expected to undergo a planned reorganization of the STEMI network. The initial inclusion period was 2 months (from 1 March until 30 April) but was subsequently prolonged to 30 June 2020. The data were compared with those retrospectively collected during the same months of 2019 (from 1 March until 30 June).

Inclusion criteria: STEMI treated with primary angioplasty (including mechanical reperfusion for failed thrombolysis).

Data Collection: Anonymized data were collected through a dedicated CRF. Each center identified a local Principal Investigator. Demographic, clinical, and procedural data, including total ischemia and door-to-balloon time, referral to primary PCI facility, COVID-19 positivity, PCI procedural data, and in-hospital mortality were recorded. Data were centralized and managed at Eastern Piedmont University.

Statistics: Data were analyzed using SPSS Statistics Software 23.0 (IBM SPSS Inc., Chicago, IL, USA) and R software (version 3.6.2) by an independent statistician (GC), as previously described [9]. Quantitative variables were described using medians and interquartile ranges. Means and confidence intervals were obtained assuming Poisson distributions for counted data. Incidence rate ratio (IRR) was defined as the ratio between the counted data in 2020 and the counted data in 2019. Data were normalized for the different sizes of national populations and for the possible different time periods of observation, and we considered the number of STEMI per million residents in the corresponding population in a year (https://knoema.com/atlas/topics/Demographics; accessed on 17 December 2022). Poisson regression models (with log link function) were applied to compare the incidence rates of primary PCIs per million residents per year in 2020 with the same rates in 2019, correcting for possible impacts of major risk factors [15]. Details are described in the Supplementary Materials (Section 1.1). Analyses were also conducted according to major European geographic areas (see Supplementary Materials) and subgroups of patients, such as age, gender, diabetes, and hypertension.

A subsequent analysis was based on individual patient data, which were grouped according to the year of the intervention (2019 vs. 2020). Absolute frequencies and percentages were used for qualitative variables. ANOVA or Mann–Whitney and chi-squared tests were used for continuous and categorical variables, respectively. Normal distribution of continuous variables was tested with the Kolmogorov–Smirnov test.

Multivariable logistic regression analyses were performed to identify the impact of the year of intervention on time delays and mortality after adjusting for baseline confounding factors between the two groups. Gender heterogeneity was assessed by the insertion of an interaction term in the regression analysis. All significant variables (set at a *p*-values < 0.1) were entered in a block into the model. Interaction was tested by the use of A, and *p* < 0.05 was considered statistically significant. The data coordination center was established at Eastern Piedmont University.

Study endpoints. The primary study endpoint was the number of primary PCIs per million residents. Secondary study endpoints were the in-hospital and 30-day mortality.

Sample size calculation. In view of the observational nature of this registry, no sample size calculations or statistical power analyses were performed.

## 3. Results

A total of 109 centers participated (Table S1), leading to the inclusion of 16,674 STEMI patients, of whom 9044 admitted in 2019 and 7630 in 2020, including 12,164 (75.6%) males and 3919 (24.4%) females.

**Figure 1:**
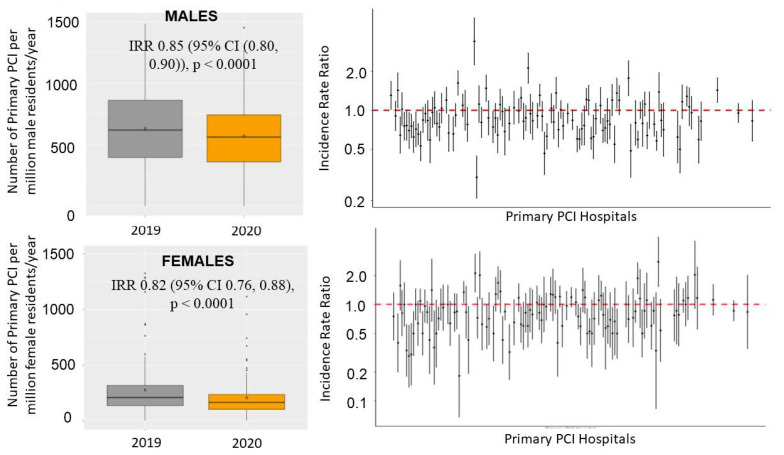
Box-and-whisker plot (on the left) showing the numbers of STEMI patients treated by mechanical reperfusion per million inhabitants/year in 2019 and 2020. The right graph shows the incidence rate ratio with 95% confidence intervals across each center.

The number of STEMI patients treated percutaneously per million residents showed a consistent reduction, on average, from 559 (95% CI 514–607) in 2019 to 477 (95% CI 435–522) in 2020. (Figure 1 and Figures S1–S3). The incidence rate ratio (IRR) was 0.843 (95% CI 0.825–0.861, *p* < 0.0001), showing a significant reduction of 15.7% in the number of STEMI cases from 2019 to 2020.

We did not find a significant gender-related reduction (*p* interaction = 0.14), with similar reductions in both sexes. Among males, the number of STEMI patients treated percutaneously per million residents had a consistent reduction, on average, from 823 (95% CI 768–881) in 2019 to 721 (95% CI 669–776) in 2020 (incidence rate ratio (IRR) of 0.852 (95% CI 0. 803, 0.903) < 0.0001) (Figure 1 and Figure S1). Significant heterogeneity was observed among the centers (IRRs had high variability among centers measured by a std error =0.28 in a random effect Poisson model; comparison with a fixed effect Poisson model provided an ANOVA chi-squared test with *p* < 0.001) (Figure 1).

Among women, the number of STEMI patients treated percutaneously per million residents had a consistent reduction, on average, from 277 (95% CI 245–312) in 2019 to 223 (95% CI 195–254) in 2020 (IRR 0.822 (95 % CI 0.764, 0.885) < 0.0001) (Figure 1 and Figure S2).

Significant heterogeneity was observed among the centers (IRRs had high variability among centers measured by a std error = 0.33 in a random effect Poisson model; comparison with a fixed effect Poisson model provided an ANOVA chi-squared test with *p* < 0.001) (Figure 1).

The heterogeneity among centers was not related to the incidence of COVID-19 infection or to COVID-related mortality (Figures S3–S6). In fact, in both genders the reduction in STEMI procedures was not associated with either of the following: the national number of COVID-19-positive patients at both 30 April (males: r = 0.065, *p*-value = 0.502; females: r = −0.108, *p*-value = 0.264) and 30 June (males r = 0.133, *p*-value = 0.169, Figure S3; females: r = −0.017, *p*-value = 0.860, Figure S4) or the national number of COVID-related deaths at 30 April (males: r = 0.031, *p*-value = 0.748; females: r = −0.133, *p*-value = 0.167) and 30 June (males: r = −0.002, *p*-value =0.983, Figure S5; females r = −0.121, *p*-value = 0.212, Figure S6). Among males, almost all participating continents had significant reductions in STEMI patients (Figures S7 and S8), whereas among females this was observed only for European centers (Figures S9 and S10). 

Furthermore, we used a Poisson regression to investigate in both male and female patients the reductions in STEMI according to subgroup, including age, hypertension, diabetes, and smoking. We found significant differences in these reductions between smokers (IRR (95% CI) = 0.819 (0.793, 0.846), *p* < 0.0001) and nonsmokers in males (IRR (95% CI) = 0.931 (0.896, 0.967), *p* < 0.0001)) (Figures S14 and S15) (*p* int < 0.0001), whereas among female patients we found a significant difference between diabetic (IRR (95% CI) = 0.916 (0.846, 0.992), *p* = 0.031) and nondiabetic patients (IRR (95% CI) = 0.792 (0.752, 0.833), *p* < 0.0001) (*p* int = 0.002) (Figures S13 and S16) and between elderly (IRR (95% CI) = 0.757 (0.700, 0.819), *p* < 0.0001) and young patients (IRR (95% CI) = 0.855 (0.812, 0.900), *p* < 0.0001) (*p* = 0.011) (Figures S11 and S16).

## 4. Baseline Demographic and Clinical Characteristics

Individual data analysis was restricted to 16,083 patients with complete demographic, clinical, procedural, and outcome data (complete cases: 96.4%), with 8698 in 2019 and 7385 in 2020. Table 1 shows the baseline characteristics of male and female patients according to year of intervention. Males treated in 2019 had a larger prevalence of smoking (*p* = 0.003) and family history of CAD (*p* = 0.034) and a different geographic distribution (*p* = 0.001) compared to 2020, whereas females treated in 2020 were younger than those treated in 2019.

**Figure 2:**
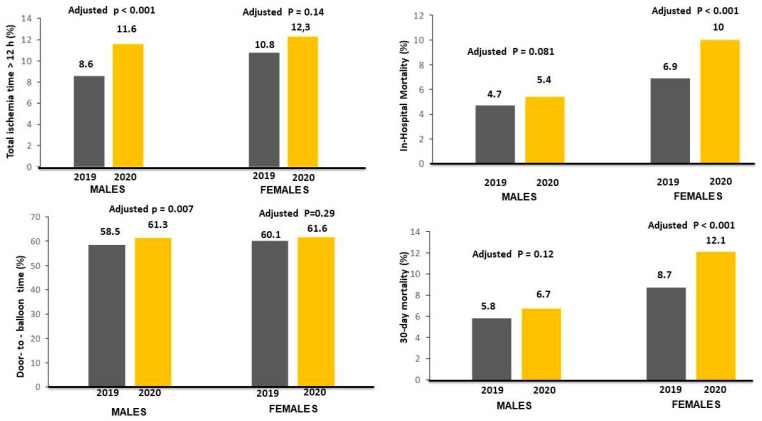
Bar graphs on the left side show the association between the year of intervention and time delays (ischemia times longer than 12 h in left upper graph; door-to-balloon times longer than 30 min in left lower graph). Bar Graphs on the right side show the association between the year of intervention with in-hospital (right upper graph) and 30-day mortality (right lower graph) rates.

As shown in Table 1, the COVID-19 pandemic was associated with longer ischemia time in both genders, whereas a significantly longer door-to-balloon time was observed only in male patients (Figure 2).

The association between the COVID-19 pandemic and ischemia times longer than 12 h was confirmed after correction for baseline clinical confounders in males (adjustment for geographic area, family history of CAD, smoking, radial access, door-to-balloon time > 30 min, additional PCI, COVID-19 positivity, and in-hospital RASI therapy; adjusted OR = 1.32 [1.12–1.55], *p* < 0.001), but not in female patients (adjustment for age, intravenous antiplatelet therapy, additional PCI, COVID-19 positivity, and in-hospital RASI therapy; adjusted OR = 1.16 [0.95–1.411, *p* = 0.14). No significant interactions were observed for major risk factors among male (age, *p* = 0.93; diabetes, *p* = 0.50; hypertension, *p* = 0.13; smoking, *p* = 0.96) and female patients (age, *p* = 0.84; diabetes, *p* = 0.52; hypertension, *p* = 0.18; smoking, *p* = 0.29).

The association between the COVID-19 pandemic and door-to-balloon times longer than 30 min was confirmed after correction for baseline clinical confounders in male patients (adjustment for geographic area, family history of CAD, smoking, radial access, ischemia time > 12 h, additional PCI, COVID-19 positivity, and in-hospital RASI therapy; adjusted OR =1.1 [1.03–1.2], *p* = 0.007). No significant interactions were observed for major risk factors among male patients (age, *p* = 0.89; diabetes, *p* = 0.87; smoking, *p* = 0.9), with the exception of hypertension (*p* = 0.022).

No differences were observed in the rates of cardiogenic shock at presentation, infarct location, out-of-hospital cardiac arrest, or rescue procedures after failed thrombolysis.

## 5. Procedural Characteristics

Concerning procedural characteristics (Table 2), the uses of DES (89.9% vs. 88.5%, *p* = 0.013) and radial access (79.3% vs. 75.5%*, p* < 0.001) were more frequent in 2020 (92.7% vs. 90.6%, *p* = 0.003) among male patients, whereas female patients had larger use of intravenous antiplatelet therapies in 2020 compared to 2019 (18.6% vs. 15.4%, *p* = 0.007). Additional in-hospital PCI and RASI therapies were observed more often in 2020 compared to 2019 in both groups.

## 6. In-Hospital and 30-Day Mortality

A significantly higher in-hospital mortality rate was observed in 2020 compared to 2019 in females (10% vs. 6.9%, OR [95% CI] = 1.44 [1.17–1.78], *p* < 0.001), but not in male patients (4.7% vs. 5.4%, OR [95% CI] = 1.14 [0.98–1.34], *p* = 0.086) (Figure 2) (*p* interaction = 0.069). The mortality rates were extremely high among COVID-19-positive patients (n = 109: 80 males and 29 females), especially among males (28.8% vs. 4.9%, *p* < 0.001) compared to female patients (17.2% vs. 8.3%*, p* = 0.081) (*p* interaction = 0.022).

These results were confirmed after correction for all potential confounding factors (female patients: adjustment for age, ischemia time, intravenous antiplatelet therapy, additional PCI, COVID-19 positivity, in-hospital RASI therapy (adjusted OR [95% CI] = 1.71 [1.34–2.18], *p* < 0.001); male patients: adjustment for geographic area, family history of CAD, smoking, radial access, door-to-balloon time > 30 min, ischemia time > 12 h, additional PCI, COVID-19 positivity, and in-hospital RASI therapy (adjusted OR [95% CI] = 1.17 [0.98-1.39], *p* = 0.081).

**Figure 3:**
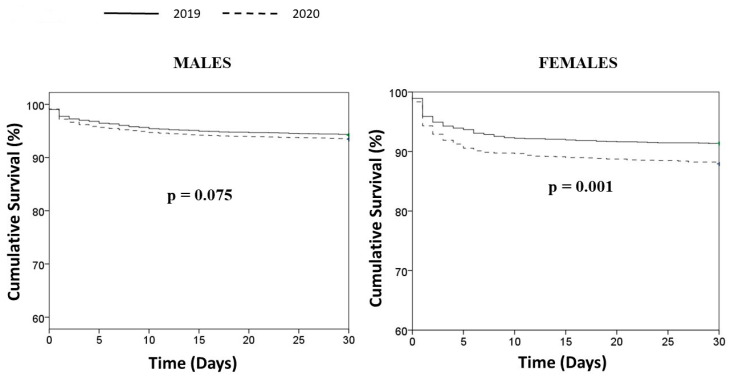
Kaplan–Meier survival curves of STEMI patients treated in 2019 and 2020.

Data on 30-day mortality were available for 14303 individuals (88.9%). Patients treated in 2020 had significantly higher mortality in female (12.1% vs. 8.7%, HR [95% CI] = 1.66 [1.31–2.11], *p* < 0.001) but not male patients (5.8% vs. 6.7%, HR [95% CI] = 1.14 [0.99–1.32], *p* = 0.074) (*p* int = 0.1). The results were confirmed after adjustment for confounders (adjusted HR [95% CI] =1.52 [1.21–1.92], *p* < 0.001; adjusted HR [95% CI] = 1.14 [0.96–1.36], *p* = 0.12, respectively) (Figure 3).

## 7. Discussion

The ISACS-STEMI COVID-19 Registry represents the largest registry worldwide, including more than 16000 STEMI patients undergoing primary PCIs during the COVID-19 pandemic treated from March to June in both 2019 and 2020, and it is the first addressing gender-related issues of the COVID-19 pandemic on the management of STEMI. Despite no gender-related difference in the number of primary PCI procedures during the pandemic period, as well as a longer time to reperfusion observed among males, the pandemic had a remarkable impact on 30-day mortality, especially among female patients.

Female gender, in fact, represents an established major determinant of negative outcomes in the setting of STEMI. Increased rates of comorbidities, more advanced age at presentation, and frailty have been generally claimed for this higher mortality, in addition to longer time to treatment [16,17,18]. Indeed, more delayed presentation and atypical symptoms have been reported more frequently among women, often preventing prompt diagnosis and management, with lower rates of primary PCI procedures. In effect, De Luca et al. clearly showed in two large cohorts of STEMI patients [19,20] that prognostic differences disappeared after correction for baseline clinical and angiographic risk profiles. In fact, female gender did not emerge as an independent predictor of mortality.

During the COVID-19 pandemic, initial reports from Chinese registries [21,22] and subsequent European cohorts, including patients treated in March and April of 2019–2020 [23,24], showed a remarkable reduction in the number of acute coronary patients presenting to hospitals due to fear of the contagion. These data were subsequently confirmed in the larger ISACS-STEMI COVID-19 Registry, including patients from Europe, America, north Africa, and Asia, although with wide variability (from −20 to −70%) compared to prepandemic data [3,4,5,21,22,23,24]. In addition, challenges in logistics due to the saturation of ambulance transportation systems and emergency departments, the shifting of healthcare resources for the management of COVID-19 patients, and the need to establish eventual positivity before admission could certainly have further contributed to prolonged ischemia time, accounting for the higher mortality of STEMI patients during this period.

Thus, an even more severe impact of delayed reperfusion or no access to invasive strategies during the COVID-19 pandemic could be expected, especially among females [25].

Moreover, hypovitaminosis D, which has emerged as a major contributor to increased mortality and cardiovascular events, in addition to favoring the susceptibility and the complications of COVID-19 infection, has been documented more frequently among females compared to males. In fact, the anti-inflammatory and antithrombotic properties of calcitriol were suggested to contrast the pathophysiological mechanisms triggered by the SARS-CoV-2 virus, as they involve endothelial damage and thrombosis, in addition to preventing the development of atherosclerosis [26].

However, despite the expected negative effects of the pandemic among the more fragile subsets of patients, including women, an opposite tendency for enhanced mortality was documented for males experiencing COVID-19 pneumonia. An early report from China have pointed to a sex imbalance with regards to the detected cases and case fatality rates of COVID-19 [27], which was confirmed even in European countries despite more contrasting data [28]. In addition, Galloway et al. [29], who analyzed over 1000 patients acutely admitted to two London hospitals with COVID-19 and positive SARS-CoV-2 swabs, identified twelve risk factors, including male gender, diabetes mellitus, hypertension, and chronic lung disease, as predictors of critical care admission and death in people admitted to hospitals with COVID-19. Indeed, higher rates of smoking, overweight patients, pre-existing cardiovascular conditions, and other risk factors and behavioral differences could favor the occurrence of respiratory and cardiovascular complications among men compared to women, where hormonal assets generally promoted immune tolerance and were more protective against thrombotic events [30].

Moreover, increasing evidence has suggested that sex and sex hormones could affect many components of the renin-angiotensin system, including a lower expression of ACE2 in women [31,32], which has been identified as a gatekeeper for the SARS-CoV-2 infection.

Thus far, few studies have compared the impact of the COVID-19 pandemic on the treatment and prognosis of STEMI among male and female patients. De Rosa et al. recently described a greater STEMI rate of decline for women than for men (41.2% vs. 17.8%) [33], although a subsequent larger study including over 2000 patients concluded that the COVID-19 pandemic period closed the gap between men and women in ACS, with similar rates of reduction in hospitalized patients. Furthermore, many typical differences between males and females regarding ischemic heart disease presentations, including more advanced age in women and vessel distribution, were leveled [34].

Similarly, Huynh et al. observed a larger reduction in the incidence of acute myocardial infarction predominantly among women aged over 70 years [35]. 

The present subanalysis of the ISACS-STEMI COVID-19 Registry conducted in high-volume primary PCI centers on several continents (Europe, Latin America, southeast Asia, and north Africa) provided relevant, reliable information to this controversial debate. We found a significant reduction in the number of STEMI patients undergoing mechanical reperfusion, with no difference according to gender. However, in the subgroup analysis, we observed a relevant interaction between male gender and smoking and greater reductions in female patients for elderly and diabetic patients, consistent with other small-sized registries [33,34].

Indeed, as previously suggested, a higher prehospital mortality rate in patients with more advanced age could condition the selection of a younger population of patients accessing primary PCI facilities.

In addition, whilst in the pre-COVID-19 era the time to reperfusion was more delayed in females, in 2020 the prolongation of the time of ischemia was more evident in males, which could be because of great fear of the contagion due to media updates on their worse prognosis for COVID-19, thus preventing their presentation to hospitals.

Moreover, atypical symptoms, including dyspnoea, could occur more frequently, leading to the misclassification of a certain proportion of STEMI patients, and COVID-19 positivity was more frequent among men, identifying a subpopulation of STEMI patients with a very severe prognosis (28.8% mortality in males vs. 17.2% among females).

Nevertheless, although the longer time to treatment contributed to explaining the overall higher mortality observed during this pandemic, as compared to 2019 in both sexes, more delayed reperfusion in males did not translate into a greater risk of mortality. In fact, female patients still maintained poorer outcomes, which was confirmed even after correction for baseline differences. Higher thrombotic burden requiring larger use of intravenous antiplatelet agents, as much as increased complexity of coronary multivessel disease, could negatively affect the prognoses of these patients.

Confirming previous reports [10,11], we observed a significantly higher impact on the mortality of SARS-CoV-2 positivity in male compared to female patients. Therefore, COVID-19 infection per se could not explain the negative global impact of the pandemic on outcomes more remarkably observed among females rather than male patients.

Nevertheless, the gender disparities observed during the COVID-19 pandemic, as well as the changes in the patterns of presentation and the management of primary PCI therapies, emphasize the need to understand the impact of sex on the incidence and pathophysiology of the disease in order to tailor treatment according to sex and gender [32].

In light of the ongoing large vaccine campaign worldwide and the developments in pharmacological drugs for the management of the infection, which have been suggested to display potentially different efficacies and side effects according to gender, based on available data, it is extremely important that scientific societies and health authorities promote public campaigns in order to highlight the importance of the prompt recognition and response to characteristic symptoms of acute myocardial infarction to positively impact the outcomes, especially among more frail categories of patients, such as those of female gender.

## 8. Limitations

As previously described [9], this study was limited by its retrospective design. It was conducted during a challenging pandemic emergency, and we expected to encounter missing data. Nevertheless, our main data analysis and conclusions were based on counts and, therefore, the overall cohort of patients was included. Furthermore, even in the analysis based on the full individual patient data, this limitation and the potential risk of type-II error was largely overcome by the high rate of complete cases (>95%) and the high statistical power due to the size of the study population. Even though in the present registry of patients undergoing mechanical reperfusion we did not find any difference in out-of-hospital cardiac arrest, we could not exclude that the reduction in STEMI patients observed in 2020 may partly result from higher rates of prehospital death due to longer delays to first medical contact, as described during the COVID-19 pandemic [9].

Finally, we could not provide any mechanistic insights to support our findings. Therefore, future additional studies are certainly needed to further investigate and unveil the potential pathophysiological mechanisms that may contribute to explaining our findings.

## 9. Conclusions

The COVID-19 pandemic had a significant impact on the treatment of patients with STEMI, with a 16% reduction in PPCI procedures similarly observed in both genders. Even though the pandemic impacted ischemia time more significantly among males, as well as SARS-CoV-2 positivity on survival, we observed significantly increased in-hospital and 30-day mortality rates during the pandemic only among females.

Therefore, gender disparities in the treatment and outcomes of STEMI patients undergoing primary PCIs are not yet overcome, and efforts should be made in order to fill in the prognostic gap.

## Figures and Tables

**Table 1:** Baseline demographic and clinical characteristics.

	2019 Males (*n* = 6571)	2020 Males (*n* = 5593)	*p*-Value	2019 Females (*n* = 2127)	2020 Females (*n* = 1792)	*p*-Value	
Age—median [IQR]	61 [53–69]	61 [53–70]	0.85	68 [59–79]	67 [58–78]	0.01 *	
Elderly (>75 y)—*n* (%)	967 (14.7)	805 (14.4)	0.624	715 (33.6)	560 (31.3)	0.615	
Medical History							
Hypertension—*n* (%)	3375 (51.4)	2896 (51.8)	0.647	1370 (64.4)	1172 (65.4)	0.517	
Diabetes mellitus—*n* (%)	1458 (22.2)	1228 (22)	0.758	580 (27.3)	546 (30.5)	0.027	
Hypercholesterolemia—*n* (%)	2591 (39.4)	2150 (38.4)	0.265	854 (40.2)	758 (42.3)	0.173	
Smoker—*n* (%)	3972 (60.4)	3233 (57.8)	0.003	857 (40.3)	724 (40.4)	0.944	
Family history of CAD—*n* (%)	1409 (21.4)	1112 (19.9)	0.034	426 (20)	351 (19.6)	0.730	
Previous STEMI—*n* (%)	680 (10.3)	575 (10.3)	0.903	152 (7.1)	136 (7.6)	0.596	
Previous PCI—*n* (%)	855 (13)	781 (14)	0.125	183 (8.6)	174 (9.7)	0.231	
Previous CABG—*n* (%)	120 (1.8)	98 (1.8)	0.759	24 (1.1)	30 (1.7)	0.144	
Geographic area			0.001			0.089	
Europe—*n* (%)	5201 (79.2)	4378 (78.3)		1782 (83.8)	1453 (81.1)		
Latin America—*n* (%)	446 (6.8)	523 (9.4)		184 (8.7)	197 (11)		
Southeast Asia—*n* (%)	613 (9.3)	506 (9)		93 (4.4)	81 (4.5)		
North Africa—*n* (%)	311 (4.7)	186 (3.3)		68 (3.2)	61 (3.4)		
*Referral to Primary PCI Hospital*							
Type							
Ambulance (from community)—*n* (%)	3111 (47.3)	2680 (47.9)	0.782	1051 (49.4)	896 (50)	0.616	
Direct access to hub—*n* (%)	1896 (28.9)	1585 (28.3)		553 (26)	479 (26.7)		
Transfer from spoke—*n* (%)	1564 (23.8)	1328 (23.7)		523 (24.6)	417 (23.3)		
*Time delays*							
Ischemia time—median [25–75th]	190 [120–340]	213 [129–390]	<0.001	215 [130–390]	240 [140–433]	<0.001 *	
Total ischemia time			<0.001			0.015	
<6 h—*n* (%)	5078 (77.3)	4063 (72.6)		1544 (72.6)	1237 (69)		
6–12 h—*n* (%)	931 (14.2)	881 (15.8)		353 (16.6)	334 (18.6)		
12–24 h—*n* (%)	376 (5.7)	417 (7.5)		161 (7.6)	134 (7.5)		
>24 h—*n* (%)	186 (2.8)	232 (4.1)		69 (3.2)	87 (4.9)		
Total ischemia time > 12 h—*n* (%)	562 (8.6)	649 (11.6)	<0.001	230 (10.8)	221 (12.3)	0.138	
Door-to-balloon time—median [25—75th]	39 [25–62]	40 [25–70]	<0.001	40 [25–70]	40 [25–70]	0.2 *	
Door-to-balloon time			<0.001			0.556	
<30 min—*n* (%)	2730 (41.5)	2165 (38.7)		849 (39.9)	689 (38.4)		
30–60 min—*n* (%)	2161 (32.9)	1836 (32.8)		684 (32.2)	578 (32.3)		
>60 min—*n* (%)	1680 (25.6)	1592 (28.5)		594 (27.9)	525 (29.3)		
Door-to-balloon time > 30 min—*n* (%)	3841 (58.5)	3428 (61.3)	0.001	1278 (60.1)	1103 (61.6)	0.349	
*Clinical Presentation*							
Anterior STEMI—*n* (%)	3036 (46.2)	2657 (47.5)	0.151	950 (44.7)	803 (44.8)	0.927	
Out-of-hospital cardiac arrest—*n* (%)	400 (6.1)	346 (6.2)	0.821	115 (5.4)	95 (5.3)	0.547	
Cardiogenic shock—*n* (%)	460 (7)	396 (7.1)	0.551	165 (7.8)	148 (8.3)	0.564	
Rescue PCI for failed thrombolysis—*n* (%)	468 (7.1)	364 (6.5)	0.181	137 (6.4)	130 (7.3)	0.314	

* Mann–Whitney test. CAD—coronary artery disease; STEMI—ST-segment elevation myocardial infarction; PCI—percutaneous coronary intervention; CABG—coronary artery bypass graft.

**Table 2:** Angiographic and procedural characteristics.

	2019 Males (*n* = 6571)	2020 Males (*n* = 5593)	*p*-Value	2019 Females (*n* = 2127)	2020 Females (*n* = 1792)	*p*-Value
Radial access—*n* (%)	4960 (75.5)	4438 (79.3)	<0.001	1563 (73.5)	1307 (72.9)	0.699
*Culprit vessel*			0.395			0.820
Left main—*n* (%)	110 (1.7)	88 (1.6)		31 (1.5)	23 (1.3)	
Left anterior descending artery—*n* (%)	3025 (46.1)	2585 (46.2)		964 (45.3)	786 (43.9)	
Circumflex—*n* (%)	967 (14.7)	867 (15.5)		279 (13.1)	237 (13.2)	
Right coronary artery—*n* (%)	2416 (36.8)	2007 (35.9)		844 (39.7)	734 (41)	
Anterolateral branch—*n* (%)	23 (0.4)	13 (0.2)		2 (0.1)	3 (0.2)	
SVG—*n* (%)	30 (0.5)	33 (0.6)		7 (0.3)	9 (0.5)	
In-stent thrombosis—*n* (%)	274 (4.2)	227 (4.1)	0.758	65 (3.1)	66 (3.7)	0.277
Vessel disease—*n* (%)			0.216			0.526
1	3311 (50.4)	2788 (49.8)		1151 (54.1)	947 (52.8)	
2	1902 (28.9)	1695 (30.3)		572 (26.9)	511 (28.5)	
3	1358 (20.7)	1110 (19.8)		404 (19)	334 (18.6)	
Preprocedural TIMI 0 flow—*n* (%)	4422 (67.3)	3784 (67.7)	0.673	1344 (63.2)	1181 (65.9)	0.077
Thrombectomy—*n* (%)	1115 (17)	921 (16.5)	0.460	287 (13.5)	240 (13.4)	0.927
Stenting—*n* (%)	6056 (92.2)	5141 (91.9)	0.620	1942 (91.3)	1627 (90.8)	0.487
Drug-eluting stent—*n* (%)	5814 (88.5)	5027 (89.9)	0.013	1842 (86.6)	1571 (87.7)	0.321
Postprocedural TIMI 3 flow—*n* (%)	6104 (92.9)	5188 (92.8)	0.775	1926 (90.6)	1603 (89.5)	0.253
Gp IIb-IIIa inhibitors/cangrelor—*n* (%)	1426 (21.7)	1180 (21.1)	0.419	327 (15.4)	334 (18.6)	0.007
Bivalirudin—*n* (%)	25 (0.4)	14 (0.3)	0.206	9 (0.4)	4 (0.2)	0.278
Mechanical support—*n* (%)	190 (2.9)	192 (3.4)	0.088	56 (2.6)	59 (3.3)	0.223
*Additional PCI*			0.009			0.046
During index procedure—*n* (%)	605 (9.2)	599 (10.7)		182 (8.6)	190 (10.6)	
Staged—*n* (%)	702 (10.7)	629 (11.2)		184 (8.7)	171 (9.5)	
DAPT therapy—*n* (%)	6492 (98.8)	5539 (99)	0.211	2101 (98.8)	1773 (98.9)	0.635
In-hospital RASI—*n* (%)	3552 (54.1)	3293 (58.9)	<0.001	1074 (50.5)	978 (54.6)	0.011
In-hospital mortality—*n* (%)	310 (4.7)	302 (5.4)	0.086	147 (6.9)	179 (10)	0.001
30-day mortality—*n* (%)	343 (5.8)	332 (6.7)	0.074	161 (8.7)	191 (12.1)	0.001

TIMI—thrombolysis in myocardial infarction; DAPT—dual antiplatelet therapy.

## Data Availability

Data are available upon request submitted to the steering committee for six months after publication.

## Supplementary Materials

The following supporting information can be downloaded at: https://www.mdpi.com/article/10.3390/jcm12030896/s1, Figure S1: Results of Poisson regression analysis in the male population to study the relationship between the number of primary PCI per million of male residents/year in 2020 vs the number in 2019; Figure S2: This graph shows the results of Poisson regression analysis in the female population to study the relationship between the number of primary PCI per million of female residents/year in 2020 vs the number in 2019; Figure S3: This graph shows in the male population the absence of significant relationship between the Incidence Rate Ratio of each centre (on the log-scaled axis) and the number of national COVID-19 cases per million of male residents. Blue lines refer to the predicted values (solid line) from Poisson model, together with 95% prediction intervals (dashed lines). Red line refers to the situation of no COVID-19 effect (intercept term); Figure S4: This graph shows in the female population the absence of significant relationship between the Incidence Rate Ratio of each centre (on the log-scaled axis) and the number of national COVID-19 cases per million of female residents. Blue lines refer to the predicted values (solid line) from Poisson model, together with 95% prediction intervals (dashed lines). Red line refers to the situation of no COVID-19 effect (intercept term); Figure S5: This graph shows in the male population the absence of significant relationship between the Incidence Rate Ratio of each centre (on the log-scaled axis) and the number of national COVID-19 related deaths per million of male residents. Blue lines refer to the predicted values (solid line) from Poisson model, together with 95% prediction intervals (dashed lines). Red line refers to the situation of no COVID-19 effect (intercept term); Figure S6: This graph shows in the female population the absence of significant relationship between the Incidence Rate Ratio of each centre (on the log-scaled axis) and the number of national COVID-19 related deaths per million of female residents. Blue lines refer to the predicted values (solid line) from Poisson model, together with 95% prediction intervals (dashed lines). Red line refers to the situation of no COVID-19 effect (intercept term); Figure S7: Box-and-whisker plot showing the number of male STEMI patients treated by mechanical reperfusion per million of male residents/year in 2019 and 2020 across 4 continents; Figure S8: Forest plots of the incidence rate ratio in the male population on the log-scaled axis with 95% confidence interval across each continent (1: Europe, 2: Latin America, 3: South East Asia, 4: North Africa); Figure S9: Box-and-whisker plot showing the number of female STEMI patients treated by mechanical reperfusion per million of female residents/year in 2019 and 2020 across 4 continents; Figure S10: Forest plots of the incidence rate ratio in the female population on the log-scaled axis with 95% confidence interval across each continent (1: Europe, 2: Latin America, 3: South East Asia, 4: North Africa); Figure S11: Box-and-whisker plot showing the number of male STEMI patients treated by mechanical reperfusion per million of male residents/year in 2019 and 2020 (left graph) and the number of female STEMI patients treated by mechanical reperfusion per million of female residents/year in 2019 and 2020 (right graph) according to age (> or <75 years); Figure S12: Box-and-whisker plot showing the number of male STEMI patients treated by mechanical reperfusion per million of male residents/year in 2019 and 2020 (left graph) and the number of female STEMI patients treated by mechanical reperfusion per million of female residents/year in 2019 and 2020 (right graph) according to hypertension; Figure S13: Box-and-whisker plot showing the number of male STEMI patients treated by mechanical reperfusion per million of male residents/year in 2019 and 2020 (left graph) and the number of female STEMI patients treated by mechanical reperfusion per million of female residents/year in 2019 and 2020 (right graph) according to diabetes; Figure S14: Box-and-whisker plot showing the number of male STEMI patients treated by mechanical reperfusion per million of male residents/year in 2019 and 2020 (left graph) and the number of female STEMI patients treated by mechanical reperfusion per million of female residents/year in 2019 and 2020 (right graph) according to smoking; Figure S15: Forest plots of the incidence rate ratio in the male population on the log-scaled axis with 95% confidence interval according to major risk factors (Diabetes, Hypertension, Age and Smoking); Figure S16: Forest plots of the incidence rate ratio in the female population on the log-scaled axis with 95% confidence interval according to major risk factors (Diabetes, Hypertension, Age and Smoking); Table S1: Characteristics of partecipating centers; Table S2: Incidence Rate Ratio (IRR) and 95% Confidence Interval in each continent and according to the type of institution.

## Abbreviations

Percutaneous coronary intervention	(PCI)
ST-segment elevation myocardial infarction	(STEMI)
Door-to-balloon time	(DTB)
Incidence rate ratio	(IRR)
Acute coronary syndrome	(ACS)
Drug-eluting stent	(DES)
Renin-angiotensin system inhibitors	(RASIs)
