# Supplementary material for: Gender Difference in the Effects of COVID-19 Pandemic on Mechanical Reperfusion and 30-Day Mortality for STEMI: Results of the ISACS-STEMI COVID-19 Registry
Source: J Clin Med. 2023 Jan 23;12(3):896. doi: 10.3390/jcm12030896 (PMC9918240; doi:10.3390/jcm12030896)
Supplement: Supplementary file 1 [file jcm-12-00896-s001.zip › jcm-2137430-supplementary.pdf]

Supplementary materials

Table of Contents

1. **Statistical analysis** ……………………………………………… **p. 3**

   1.1 Poisson Regression analysis………………………………….. **p. 4**

2. **Supplemetary Tables**………………..…………………………….. **p. 5**

3. **Supplementary Figure Legends**…………………………………. **p. 20**

4. **Supplementary Figures**…………………………………………….. **p. 23**

1. STATISTICAL ANALYSIS.

1.1. Poisson Regression models.

Poisson regression models (with log link function) were applied to compare the Incidence rate of Primary PCI per million of residents per-year in 2020 with the same rate in 2019, correcting for possible impact of risk factors

A first Poisson model assumed the response variable to be the primary PCI count data (per million of residents per-year) in 2020 by Center, including two offset terms to accounts for the different size of the population in the Province where the Center is located, and the primary PCI count data in 2019. In this model, explanatory variables related to risk factors were constructed by aggregating information by Center in 2020, as compared to 2019, and were obtained by percentage ratios (E.g.: percentage of elderly (> 75 years) in 2020 / percentage of elderly (> 75 years) in 2019; percentage of diabetes in 2020 / percentage of diabetes in 2019; percentage of Hypertension 2020 / percentage of Hypertension in 2019).

To study the incidence rate ratio of primary PCI between 2020 and 2019, in specific subgroups of the populations, such as elderly (≥ 75 years) and young (< 75 years), diabetic and non diabetic patients, smokers and non smokers, subjects with or without hypertension, Poisson regression used as response variable, the number of primary PCI in 2020 of each subgroup in each Center, and included offsets to account for the different population sizes and the corresponding number in each subgroup in 2019.

Model adequacy and goodness of fit were performed via a residual analysis. Poisson regression was implemented by the R software (version 3.6.2).

2.0 Supplementary Tables

Table 1S. Characteristics of partecipating centers.

Table 1S. Characteristics of partecipating centers.

Center	Country	Type Institution	Total PCI 2019	Total Primary PCI 2019
Ospedale degli Infermi, Biella	Italy	Non Academic Public Hospital	451	122
Azienda Ospedaliero-Universitaria “Maggiore della Carità”, Novara	Italy	Academic Public Hospital	1076	272
Azienda Ospedaliero-Universitaria “SS. Annunziata” Sassari	Italy	Academic Public Hospital	664	179
Ospedale “San Giovanni di Dio e Ruggi d'Aragona”, Salerno	Italy	Academic Public Hospital	859	220
Ospedale "A. Manzoni" Lecco	Italy	Non Academic Public Hospital	644	141
Ospedale “Santa Maria delle Grazie”, Pozzuoli, Napoli	Italy	Non Academic Public Hospital	730	260

Ospedale “G. Moscati”, Aversa, Caserta	Italy	Non Academic Public Hospital	557	176
Ospedale “Santa Maria della Misericordia”, Perugia	Italy	Academic Public Hospital	1213	377
Ospedale “Santa Chiara”, Trento	Italy	Non Academic Public Hospital	1033	304
Azienda Ospedaliero - Universitaria “Ospedali Riuniti” Trieste	Italy	Academic Public Hospital	723	206
Ospedale “Santa Maria Goretti”, Latina	Italy	Non Academic Public Hospital	1057	509
Clinica “Villa dei Fiori”, Acerra, Napoli	Italy	Private Hospital	808	330
Ospedale “Santa Maria della Misericordia”, Udine	Italy	Academic Public Hospital	598	240
Bristol Heart Institute, University Hospitals Bristol NHSFT & University of Bristol, Bristol	UK	Academic Public Hospital	2088	737

“John Radcliffe” Hospital, Oxford	UK	Academic Public Hospital	1261	350
University Hospital Brno, Medical Faculty of Masaryk University Brno	Czech Republic	Academic Public Hospital	1250	319
Department of Cardiology, Medical Center Ljubljana, Slovenia	Slovenia	Academic Public Hospital	2114	760
Hospital “la Paz”, Madrid	Spain	Academic Public Hospital	1102	341
Hospital “Puerta del Mar”, Cadiz	Spain	Academic Public Hospital	867	163
Complejo Hospitalario de Toledo, Toledo	Spain	Academic Public Hospital	875	289
University Hospital “Juan Ramón Jiménez”, Huelva	Spain	Academic Public Hospital	1005	296
University Hospital of Wales Cardiff	UK	Academic Public Hospital	1823	552

Hospital Clínico Universitario “Virgen de la Victoria”, Málaga	Spain	Academic Public Hospital	1421	359
Complexo Hospetaliero Universitario La Coruna, La Coruna	Spain	Academic Public Hospital	1197	286
Onze Lieve Vrouwe Gasthuis (OLVG), Amsterdam	The Netherlands	Non Academic Public Hospital	2109	242
Radboud University Medical Centre Nijmegen	The Netherlands	Academic Public Hospital	1687	219
Maastricht University Medical Center	The Netherlands	Academic Public Hospital	751	379
Cardiology Maasstad Ziekenhuis, Rotterdam	The Netherlands	Non Academic Public Hospital	1752	340
University Hospital Munich, “Ludwig-Maximilians University”, Munich	Germany	Academic Public Hospital	741	240
Medical University of Silezia, Katowice	Poland	Academic Public Hospital	2472	568

St-Jan Hospital, Brugge	Belgium	Non Academic Public Hospital	1300	190
Jessa Ziekenhuis, Hasselt	Belgium	Non Academic Public Hospital	1522	189
Groupe Hospitalier Mutualiste de Grenoble	France	Private Hospital	1346	150
CHU Lariboisière, AP-HP, Paris VII University, INSERM UMRS 942	France	Academic Public Hospital	1135	147
CHU Timone, Marseille, France; Faculté de Médecine, Aix-Marseille Université, Marseille	France	Academic Public Hospital	906	215
Center Hospitalier Universitaire de Poitiers, Poitiers, University Hospital	France	Academic Public Hospital	1768	185
Azienda Ospedaliero Universitaria “Ospedali Riuniti”, Ancona	Italy	Academic Public Hospital	1031	331
Ziekenhuis Netwerk Antwerpen (ZNA) Middelheim, Antwerp	Belgium	Non Academic Public Hospital	1564	164

Ospedale “F. Spaziani“, Frosinone	Italy	Non Academic Public Hospital	480	254
Ospedale ”S. Maurizio” Bolzano	Italy	Non Academic Public Hospital	935	335
Ospedale “Sant’Anna”, Ferrara	Italy	Academic Public Hospital	2092	495
Ospedale Civico “Arnas”, Palermo	Italy	Non Academic Public Hospital	873	222
Azienda Ospedaliera “Ospedali Riuniti Marche Nord”, Pesaro	Italy	Non Academic Public Hospital	1191	239
University Hospital, Dijon	France	Academic Public Hospital	1620	311
University Hospital, Prague	Czech Republic	Academic Public Hospital	982	200
Hospital “Cabueñes”, Gijon	Spain	Academic Public Hospital	730	260

St Antonius Hospital, Nieuwegein	The Netherlands	Non Academic Public Hospital	1899	398
University Central Hospital, Helsinki	Finland	Academic Public Hospital	1795	477
Hospital Clinico Universitario, Valencia	Spain	Academic Public Hospital	684	178
Hospital Germans Triasi Pujol, Badalona	Spain	Academic Public Hospital	1003	375
Hospital Universitario de Canarias, Santa Cruz de Tenerife	Spain	Academic Public Hospital	539	176
University Hospital, Oulu	Finland	Academic Public Hospital	1095	165
H. Universitario y Politécnico “La Fe”, Valencia	Spain	Academic Public Hospital	872	376
Ospedale Maggiore Bologna	Italy	Non Academic Public Hospital	1119	342

AUSL-IRCCS Reggio Emilia	Italy	Non Academic Public Hospital	822	271
Clinical and Experimental Interventional Cardiology, University of Saarland	Germany	Academic Public Hospital	931	210
Ospedale “del Mare”, Napoli	Italy	Non Academic Public Hospital	1206	486
UMC Utrecht	The Netherlands	Academic Public Hospital	1450	275
Hospital “Puerta de Hierro”, Majadahonda	Spain	Academic Public Hospital	777	156
Central Hospital of Medical University, Lodz	Poland	Academic Public Hospital	933	189
Azienda Ospedaliera Sanitaria, Parma	Italy	Academic Public Hospital	667	203
Universitets Hospital, Odense	Danemark	Academic Public Hospital	2274	629

Northwest Clinics, Alkmaar	The Netherlands	Non Academic Public Hospital	1561	339
Heart Disease Institute, Hospital Universitari de Bellvitge Barcelona	Spain	Academic Public Hospital	1787	510
State Research Institute for Complex Issues of Cardiovascular Diseases, Kemerovo	Russia	Academic Public Hospital	3200	540
Department of Cardiology, Medisch Spectrum, Enschede	The Netherlands	Non Academic Public Hospital	1659	458
University Clinic for Cardiology, Medical Faculty, “Ss' Cyril and Methodius” University, Skopje	Macedonia	Academic Public Hospital	2800	840
Center for Cardiovascular Diseases, Ohrid	Macedonia	Private Hospital	679	361
Hospital de Santa Cruz, CHLO - Carnaxide, Portugal	Portugal	Academic Public Hospital	943	201
Ospedali Riuniti, Reggio Calabria	Italy	Non Academic Public Hospital	934	283

Clinic Emercency Hospital, Bucharest	Romania	Academic Public Hospital	1350	750
Attikon University Hospital, Athens,	Greece	Academic Public Hospital	547	190
University Hospital, Birmingham	UK	Academic Public Hospital	784	231
Heart Center,Turku	Finland	Academic Public Hospital	1068	176
Amphia Hospital, Breda	The Netherlands	Non Academic Public Hospital	2069	885
Invasive Cardiology and Congenital Heart Disease University Hospital, Patras	Greece	Academic Public Hospital	960	389
Homolka Hospital, Prague	Czech Republic	Non Academic Public Hospital	855	200
Otamendi Hospital, Buenos Aires, Argentina	Argentina	Private Hospital	1008	120

University Hospital Centre, University of Zagreb, Zagreb, Croatia	Croatia	Academic Public Hospital	1200	400
Instituto Cardiovascular de Buenos Aires, Buenos Aires, Argentina	Argentina	Private Hospital	1955	178
Center Hospitalier Universitaire de Lille, Lille, France	France	Academic Public Hospital	1097	150
Cardiology Institute, Instambul University, Instambul, Turkey	Turkey	Academic Public Hospital	980,0	180
Eskisehir Osmangazi University, Faculty of Medicine, Eskisehir, Turkey	Turkey	Academic Public Hospital	950	150
Tyumen Cardiology Research Center, Tyumen Russia	Russia	Academic Public Hospital	905	150
Universidad UPB, Universidad CES. Medellin, Columbia	Columbia	Academic Public Hospital	850,0	145
Instituto de Cardiologia Integral, Montevideo	Uruguay	Private Hospital	566	160

Assiut University, Assiut	Egypt	Academic Public Hospital	2520	1006
National University Hospital, Singapore	Singapore	Academic Public Hospital	1607	520
Queen Mary Hospital, University of Hong Kong,	HongKong	Academic Public Hospital	756	128
Queen Elizabeth Hospital, University of Hong Kong	HongKong	Academic Public Hospital	1261	322
University of Indonesia National Cardiovascular Center “Harapan Kita”, Jakarta	Indonesia	Academic Public Hospital	1185	557
Instituto de Cardiología de Corrientes "Juana F. Cabral, Corrientes	Argentina	Academic Public Hospital	1058	134
National Heart Center, Singapore	Singapore	Academic Public Hospital	2910	495
Bursa Sehir Hospital, Bursa	Turkey	Academic Public Hospital	3500	1570

Pelita Harapan University and Heart center Siloam Hospital Lippo Village, Tangerang, Indonesia	Indonesia	Academic Public Hospital	487	130
National Taiwan University Hospital Taipei, Taiwan	Taiwan	Academic Public Hospital	1586	177
Hospital of Antibes Juan Les Pins, Antibes	France	Academic Public Hospital	858	268
InstitutoNacional de CirugíaCardíaca. Montevideo, Uruguay	Uruguay	Private Hospital	609	131
Alexandra Hospital, Athens, Greece,	Greece	Academic Public Hospital	579	207
Kontantopoulion Hospital, Athens, Greece	Greece	Non Academic Public Hospital	662	178
Instituto de cardiologia do Rio Grande do Sul, Porto Alegre	Brasil	Private Hospital	3673	442
Hospital de Santo António, Porto	Portugal	Academic Public Hospital	769	244

Hospital Garcia de Orta, Almada	Portugal	Non Academic Public Hospital	659	160
Blida University Hospital, Blida	Algeria	Academic Public Hospital	1285	164
Iraklion University Hospital,Crete,	Greece	Academic Public Hospital	992	132
Instituto de Cardiologia de Santa Catarina Praia Comprida, São José	Brasil	Academic Public Hospital	986	272
Centro PROCAPE, Federal University of Pernambuco, Recife	Brasil	Academic Public Hospital	1576	797
Hospital Bezmialem Vakıf University İstanbul	Turkey	Academic Public Hospital	1000	195
Hospital Cordoba, Cordoba	Argentina	Academic Public Hospital	436	133

Table 2S. Incidence Rate Ratio (IRR) and 95% Confidence Interval in each continent and according to the type of institution.

Variable	*IRR (2020/2019)*	*95% Conf. Interval*	*p-value*
**ELDERLY**			
Overall	0.825	(0.8-0.851)	<0.001
Europe	0.769	(0.722-0.819)	<0.001
Latin America	0.955	(0.848, 1.077)	0.455
South East Asia	1.004	(0.888, 1.134)	0.952
North-Africa	0.840	(0.717,0984)	0.031
Type institution (Private vs Public Academic)	1.070	(0.925, 1.239)	0.363
Type institution (Public Non-Academic vs Public Academic)	1.029	(0.953, 1.112)	0.463
**YOUNG**			
Overall	0.861	(0.832, 0.89)	< 0.001
Europe	0.804	(0.752-0.860)	< 0.001
Latin-America	0.892	(0.775, 1.027)	0.212
South-East Asia	0.737	(0.648, 0.838)	< 0.001
North-Africa	0.464	(0.379-0.578)	< 0.001
Type institution (Private vs Public Academic)	0.907	(0.785, 1.050)	0.191
Type institution (Public Non-Academic vs Public Academic)	0.958	(0.881, 1.042)	0.879

.0 Figure Legend

Figure 1S. This graph shows the results of Poisson regression analysis in the male population to study the relationship between the number of primary PCI per million of male residents/year in 2020 vs the number in 2019.

Figure 2S. This graph shows the results of Poisson regression analysis in the female population to study the relationship between the number of primary PCI per million of female residents/year in 2020 vs the number in 2019.

Figure 3S. This graph shows in the male population the absence of significant relationship between the Incidence Rate Ratio of each centre (on the log-scaled axis) and the number of national Covid-19 cases per million of male residents. Blue lines refer to the predicted values (solid line) from Poisson model, together with 95% prediction intervals (dashed lines). Red line refers to the situation of no Covid-19 effect (intercept term).

Figure 4S. This graph shows in the female population the absence of significant relationship between the Incidence Rate Ratio of each centre (on the log-scaled axis) and the number of national Covid-19 cases per million of female residents. Blue lines refer to the predicted values (solid line) from Poisson model, together with 95% prediction intervals (dashed lines). Red line refers to the situation of no Covid-19 effect (intercept term).

Figure 5S. This graph shows in the male population the absence of significant relationship between the Incidence Rate Ratio of each centre (on the log-scaled axis) and the number of national Covid-19 related deaths per million of male residents. Blue lines refer to the predicted values (solid line) from Poisson model, together with 95% prediction

intervals (dashed lines). Red line refers to the situation of no Covid-19 effect (intercept term).

Figure 6S. This graph shows in the female population the absence of significant relationship between the Incidence Rate Ratio of each centre (on the log-scaled axis) and the number of national Covid-19 related deaths per million of female residents. Blue lines refer to the predicted values (solid line) from Poisson model, together with 95% prediction intervals (dashed lines). Red line refers to the situation of no Covid-19 effect (intercept term).

Figure 7S. Box-and-whisker plot showing the number of male STEMI patients treated by mechanical reperfusion per million of male residents/year in 2019 and 2020 across 4 continents.

Figure 8S. Forest plots of the incidence rate ratio in the male population on the log-scaled axis with 95% confidence interval across each continent (1: Europe, 2: Latin America, 3: South East Asia, 4: North Africa).

Figure 9S. Box-and-whisker plot showing the number of female STEMI patients treated by mechanical reperfusion per million of female residents/year in 2019 and 2020 across 4 continents.

Figure 10S. Forest plots of the incidence rate ratio in the female population on the log-scaled axis with 95% confidence interval across each continent (1: Europe, 2: Latin America, 3: South East Asia, 4: North Africa).

Figure 11S. Box-and-whisker plot showing the number of male STEMI patients treated by mechanical reperfusion per million of male residents/year in 2019 and 2020 (left graph) and

the number of female STEMI patients treated by mechanical reperfusion per million of female residents/year in 2019 and 2020 (right graph) according to age (≥ or < 75 years)

Figure 12S. Box-and-whisker plot showing the number of male STEMI patients treated by mechanical reperfusion per million of male residents/year in 2019 and 2020 (left graph) and the number of female STEMI patients treated by mechanical reperfusion per million of female residents/year in 2019 and 2020 (right graph) according to hypertension.

Figure 13S. Box-and-whisker plot showing the number of male STEMI patients treated by mechanical reperfusion per million of male residents/year in 2019 and 2020 (left graph) and the number of female STEMI patients treated by mechanical reperfusion per million of female residents/year in 2019 and 2020 (right graph) according to diabetes.

Figure 14S. Box-and-whisker plot showing the number of male STEMI patients treated by mechanical reperfusion per million of male residents/year in 2019 and 2020 (left graph) and the number of female STEMI patients treated by mechanical reperfusion per million of female residents/year in 2019 and 2020 (right graph) according to smoking.

Figure 15S. Forest plots of the incidence rate ratio in the male population on the log-scaled axis with 95% confidence interval according to major risk factors (Diabetes, Hypertension, Age and Smoking).

Figure 16S. Forest plots of the incidence rate ratio in the female population on the log-scaled axis with 95% confidence interval according to major risk factors (Diabetes, Hypertension, Age and Smoking).

Figure 1S

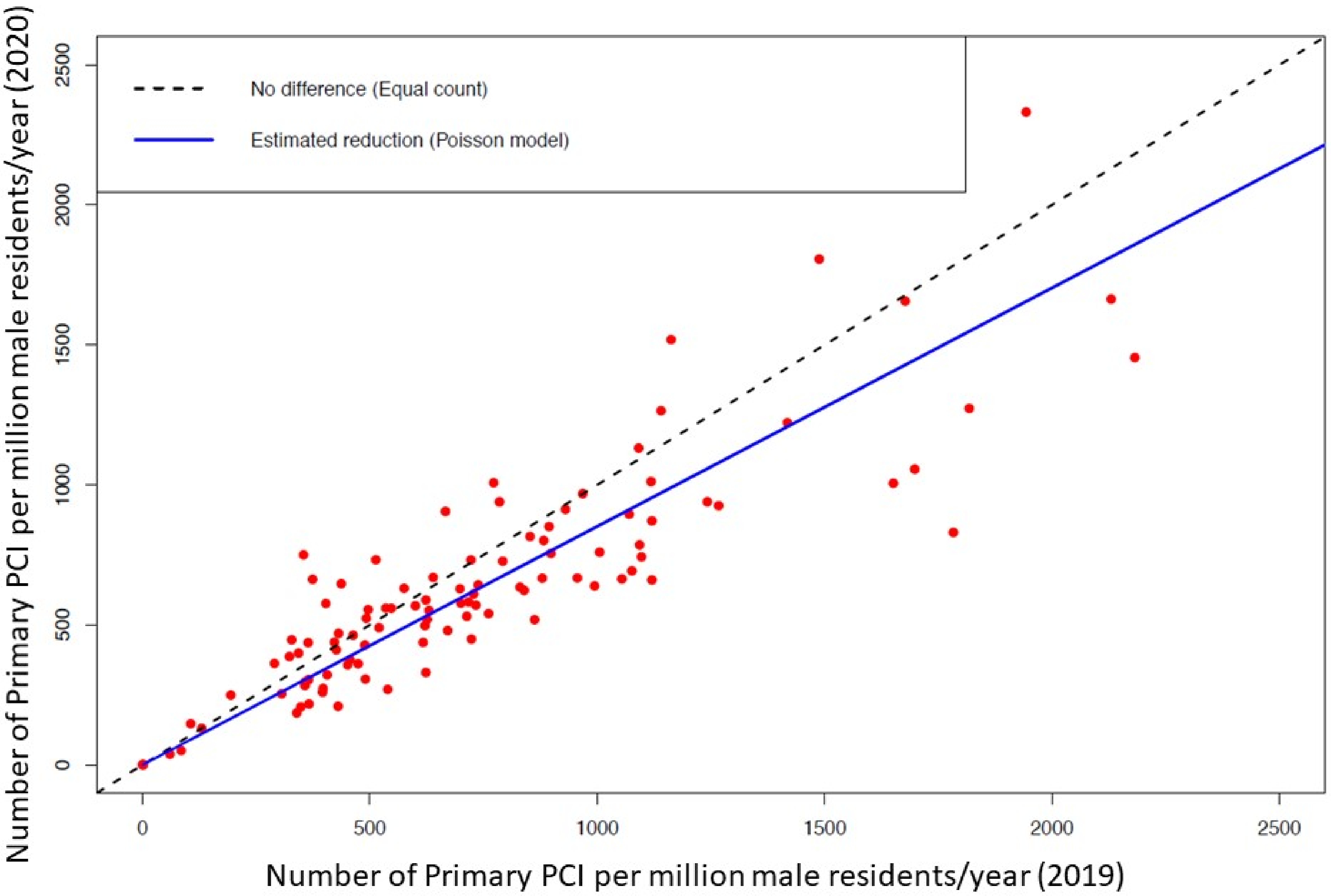

No difference (Equal count)
Estimated reduction (Poisson model)
Number of Primary PCI per million male residents/year (2020)
Number of Primary PCI per million male residents/year (2019)
0
500
1000
1500
2000
2500

Figure 2S

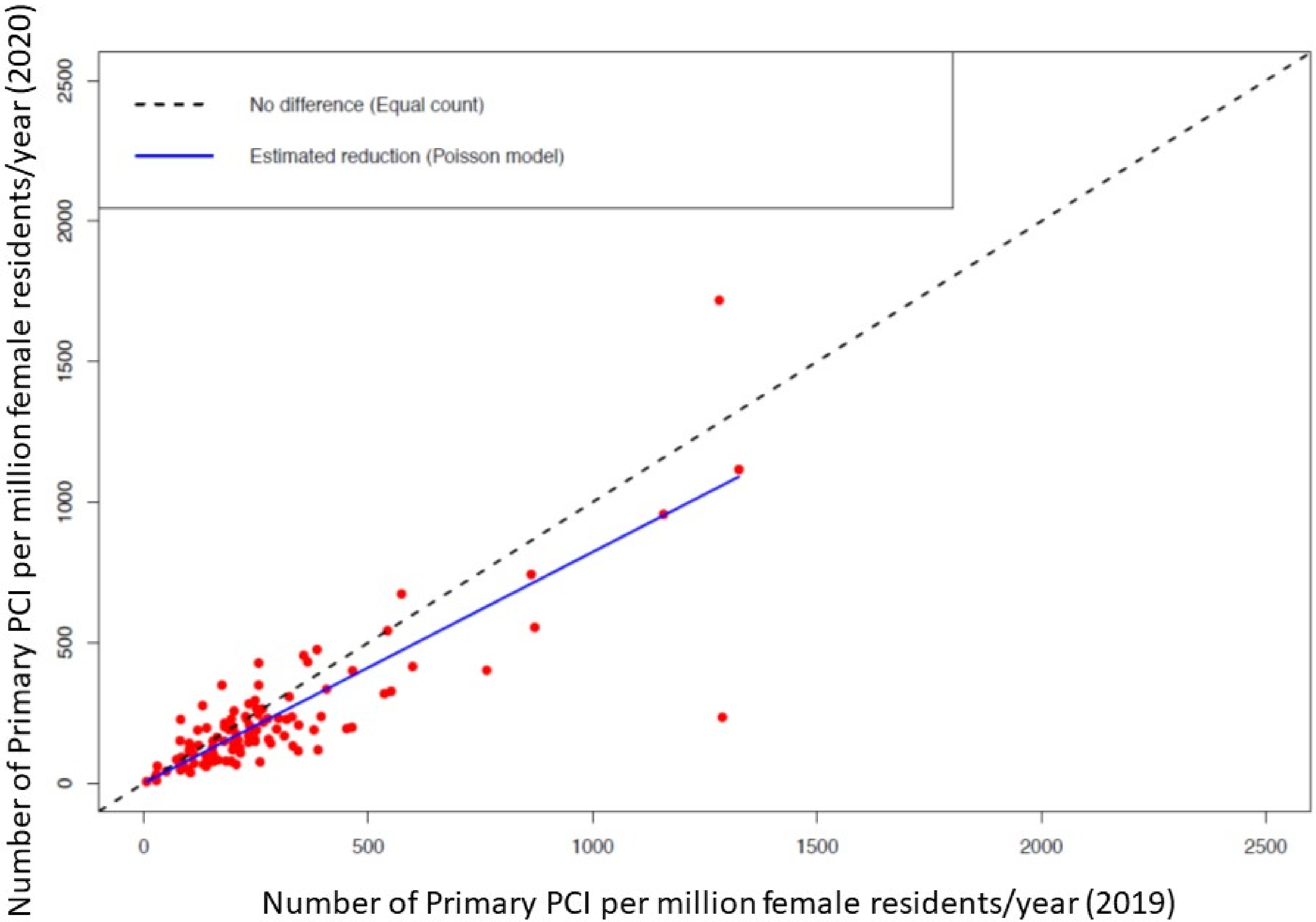
No difference (Equal count)
Estimated reduction (Poisson model)
0
500
1000
1500
2000
2500
Number of Primary PCI per million female residents/year (2020)
Number of Primary PCI per million female residents/year (2019)

Figure 3S

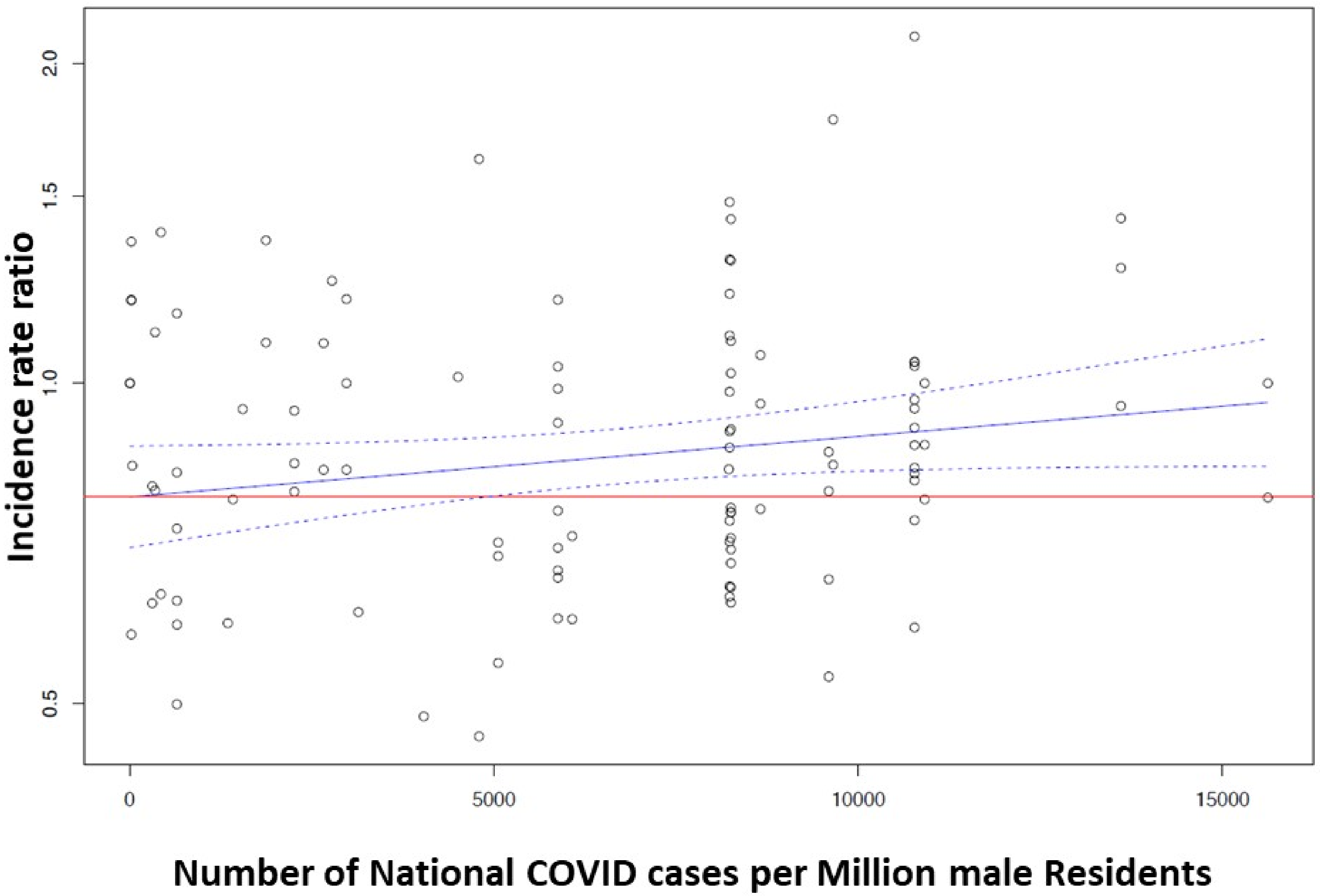

Incidence rate ratio
2.0
1.5
1.0
0.5
0
5000
10000
15000
Number of National COVID cases per Million male Residents

Figure 4S

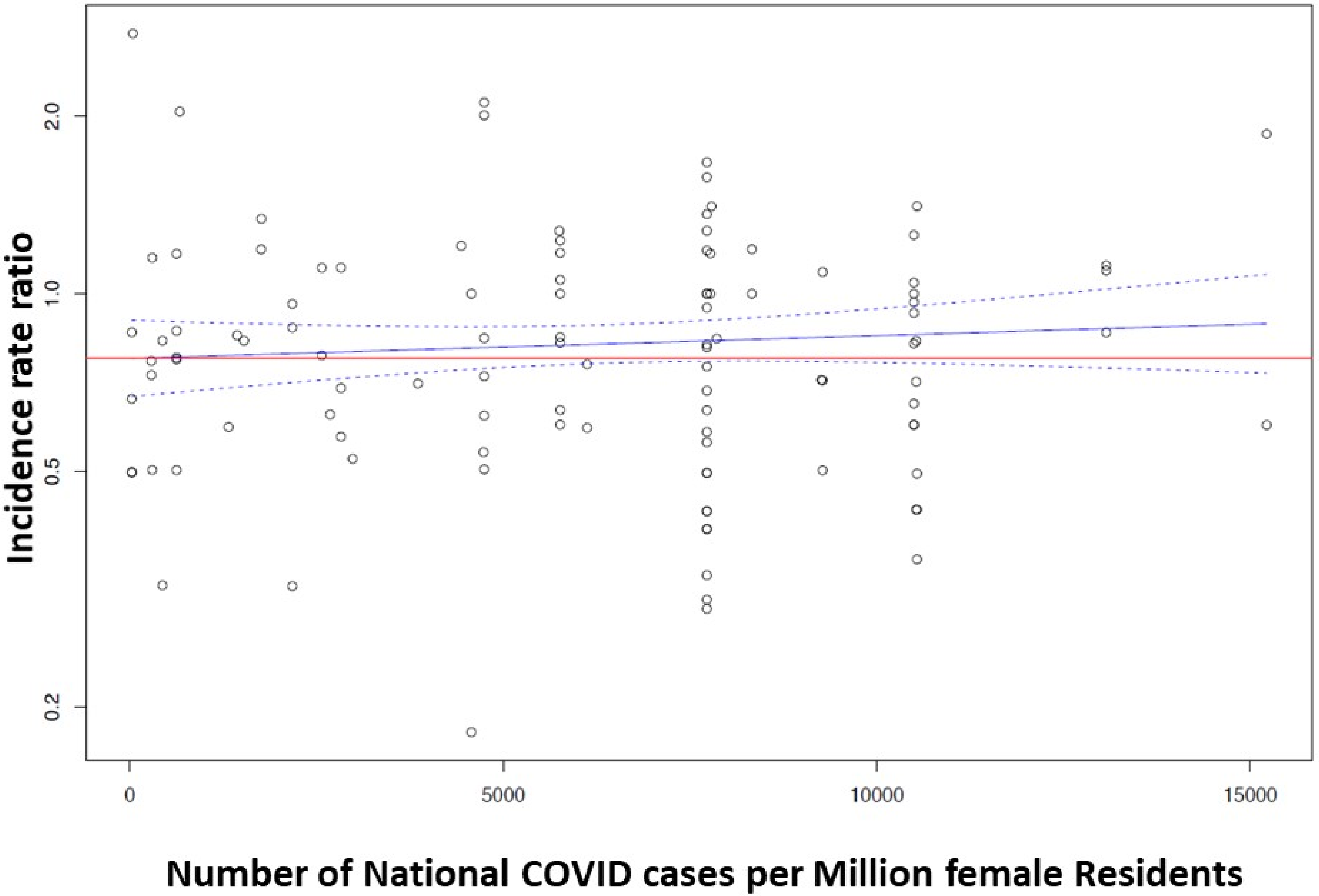

Incidence rate ratio
2.0
1.0
0.5
0.2
0
5000
10000
15000
Number of National COVID cases per Million female Residents

Figure 5S

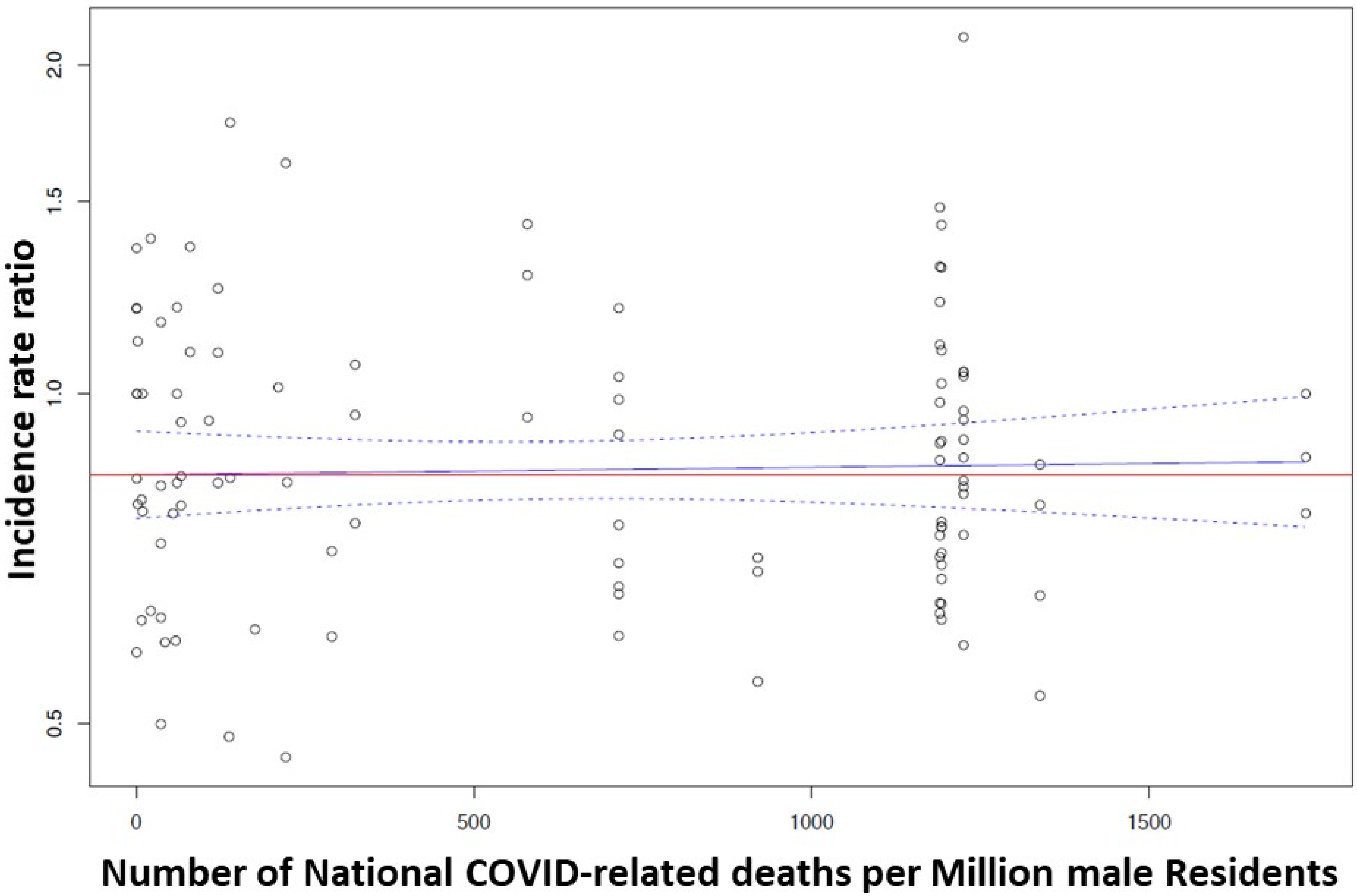
Incidence rate ratio
2.0
1.5
1.0
0.5
0
500
1000
1500
Number of National COVID-related deaths per Million male Residents

Figure 6S

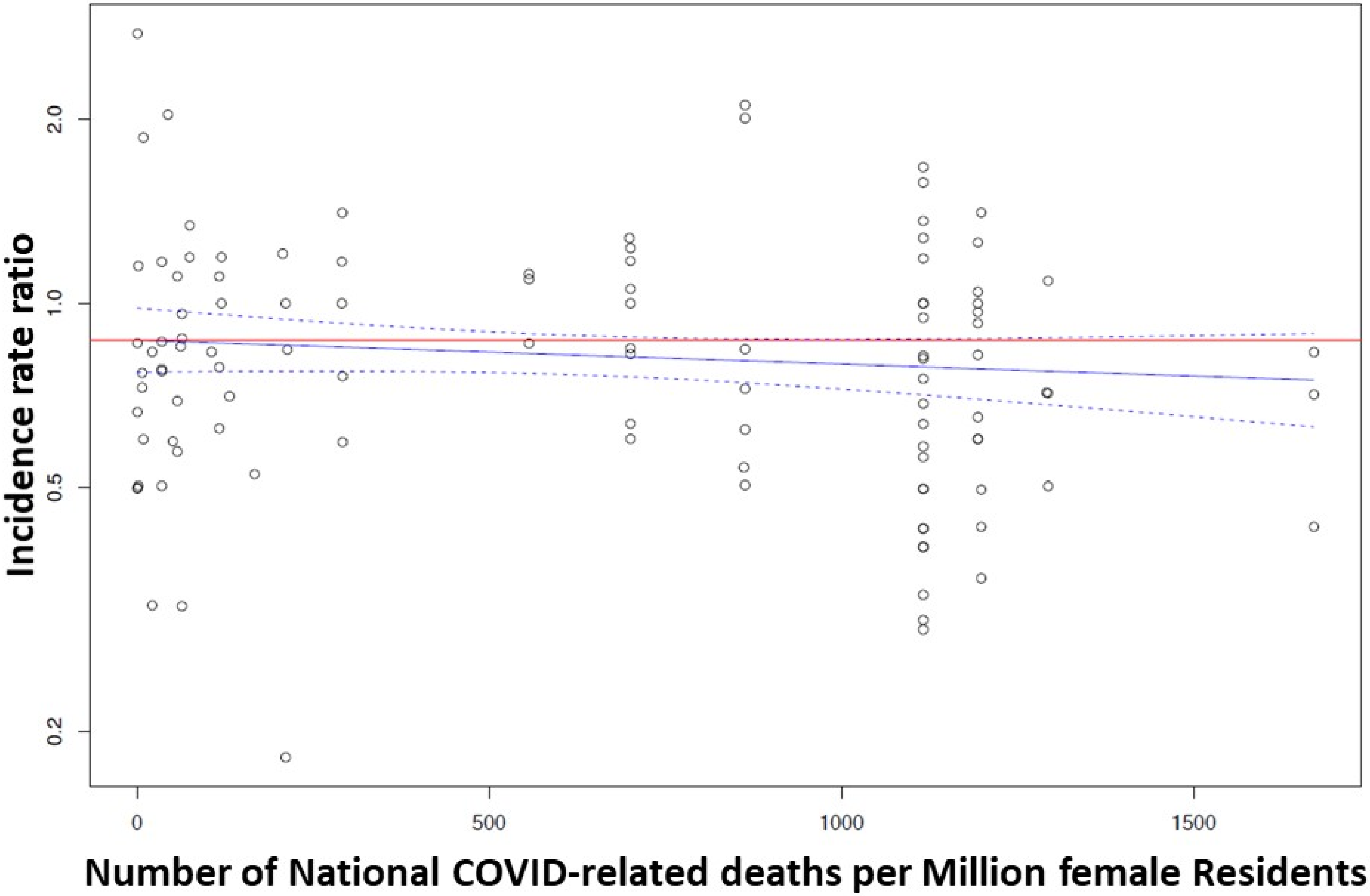

Incidence rate ratio
0.2
0.5
1.0
2.0
0
500
1000
1500
Number of National COVID-related deaths per Million female Residents

Figure 7S

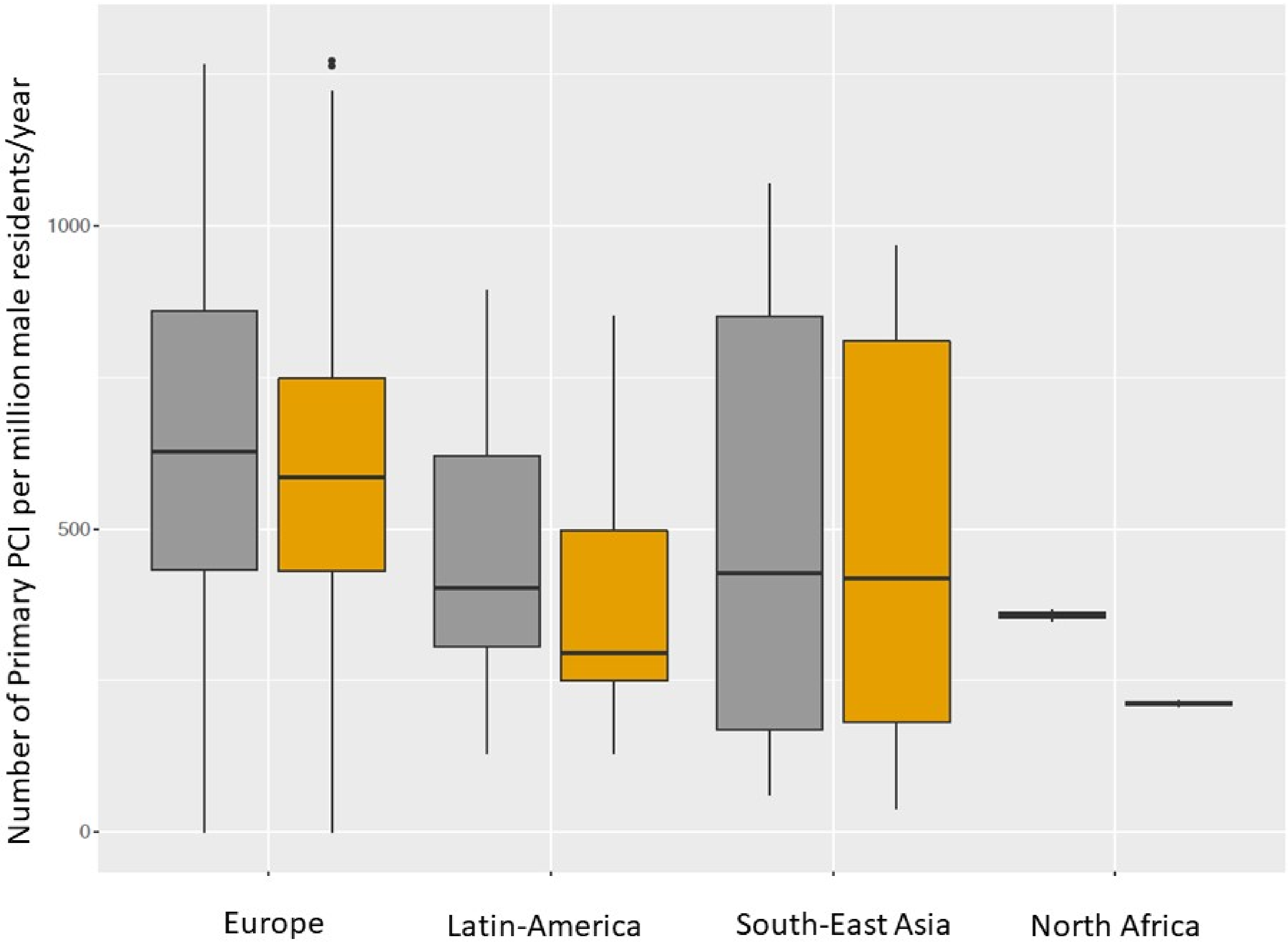
Number of Primary PCI per million male residents/year
1000
500
0
Europe
Latin-America
South-East Asia
North Africa

Figure 8S

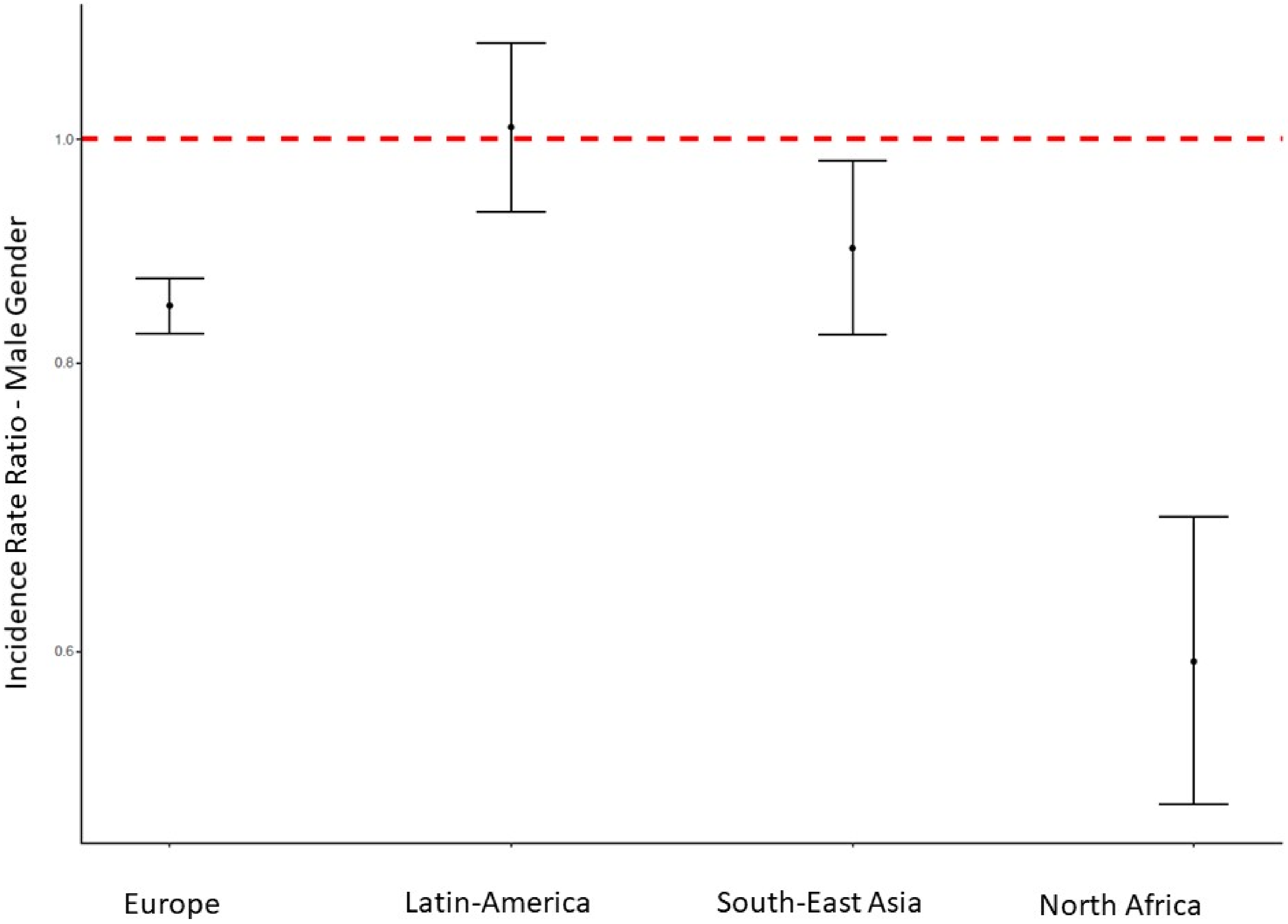
Incidence Rate Ratio - Male Gender
1.0
0.8
0.6
Europe
Latin-America
South-East Asia
North Africa

Figure 9S

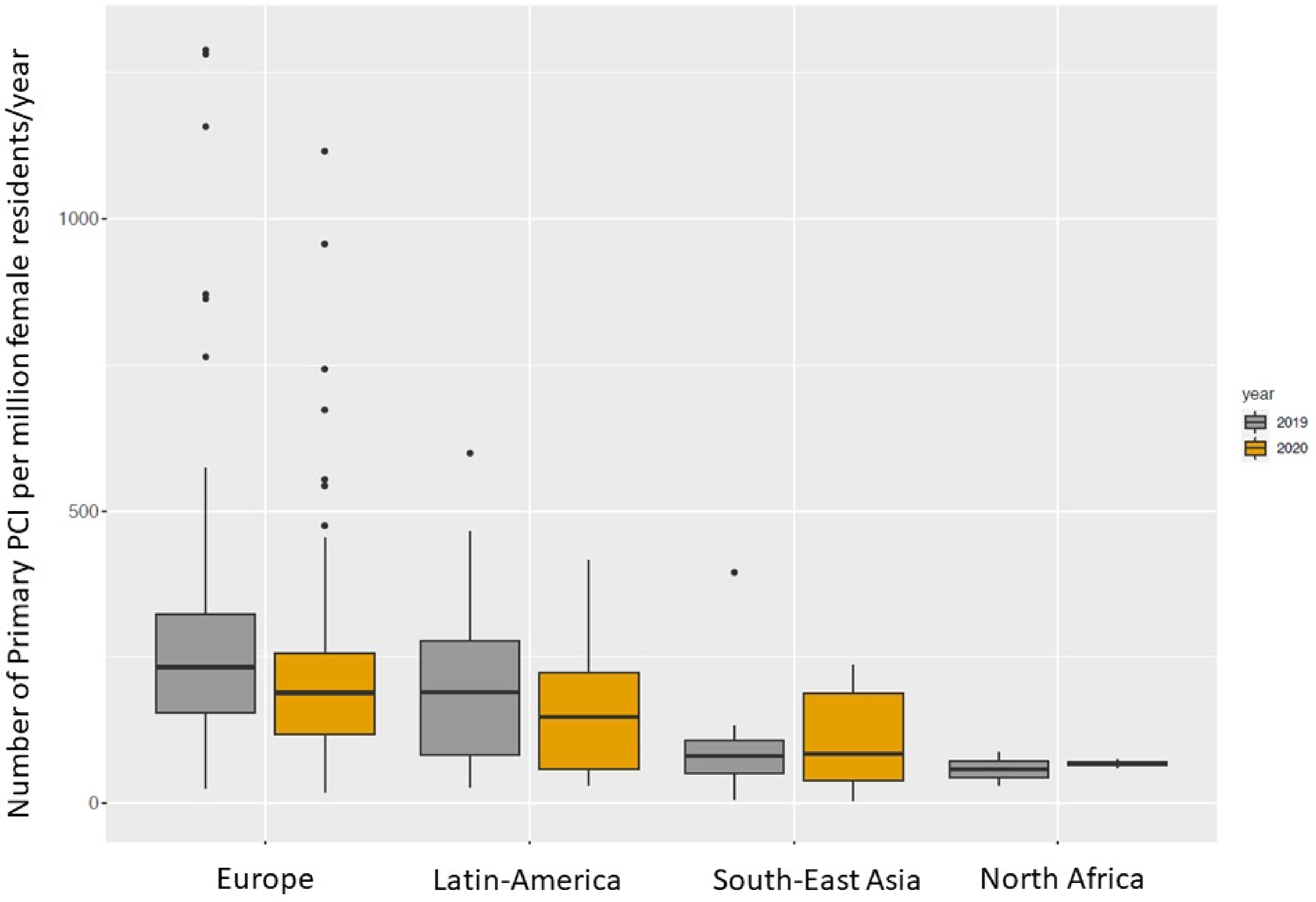
Number of Primary PCI per million female residents/year
1000
500
0
Europe
Latin-America
South-East Asia
North Africa
year
2019
2020

Figure 10S

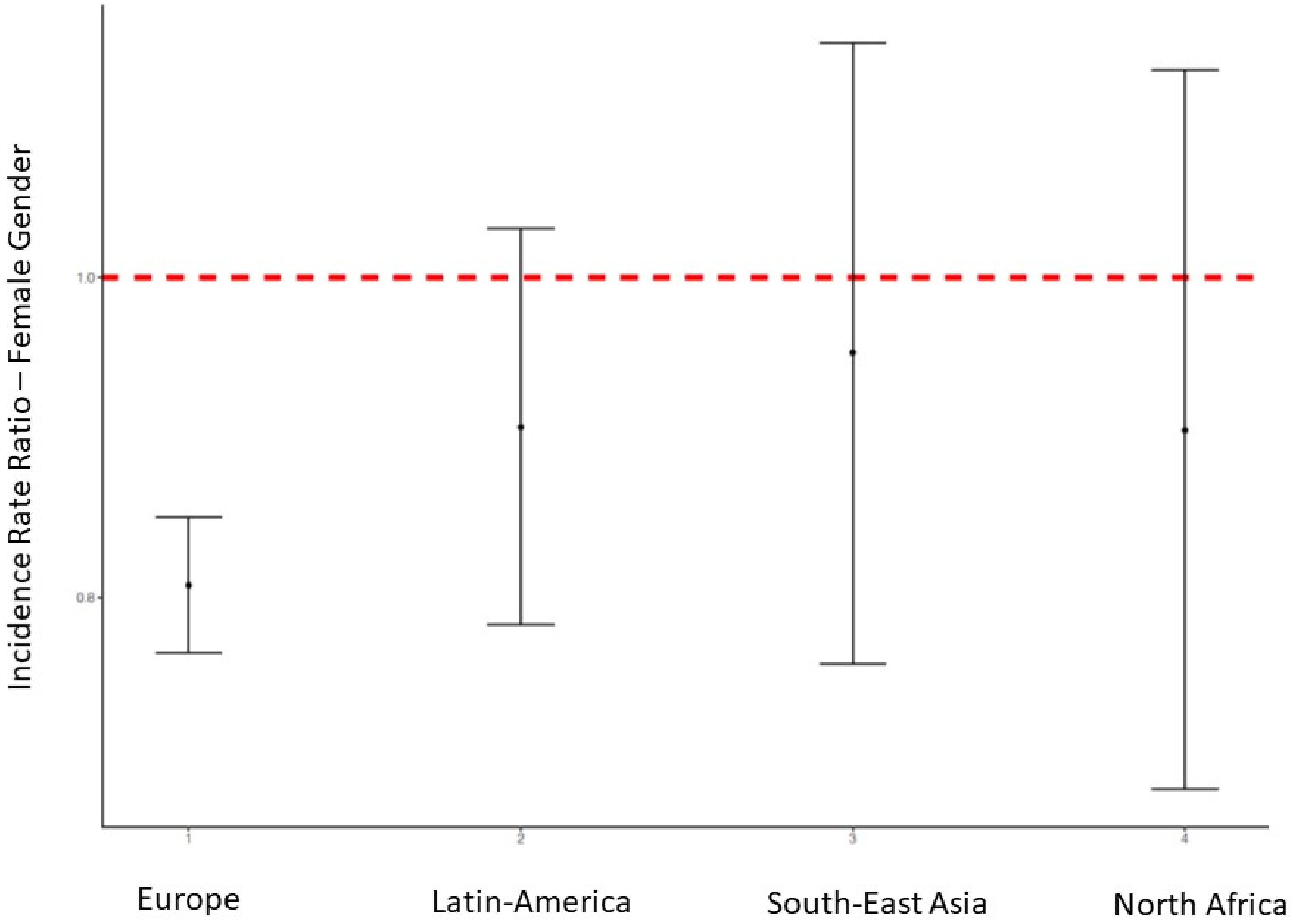

Incidence Rate Ratio – Female Gender
1.0
0.8
1
2
3
4
Europe
Latin-America
South-East Asia
North Africa

Figure 11S

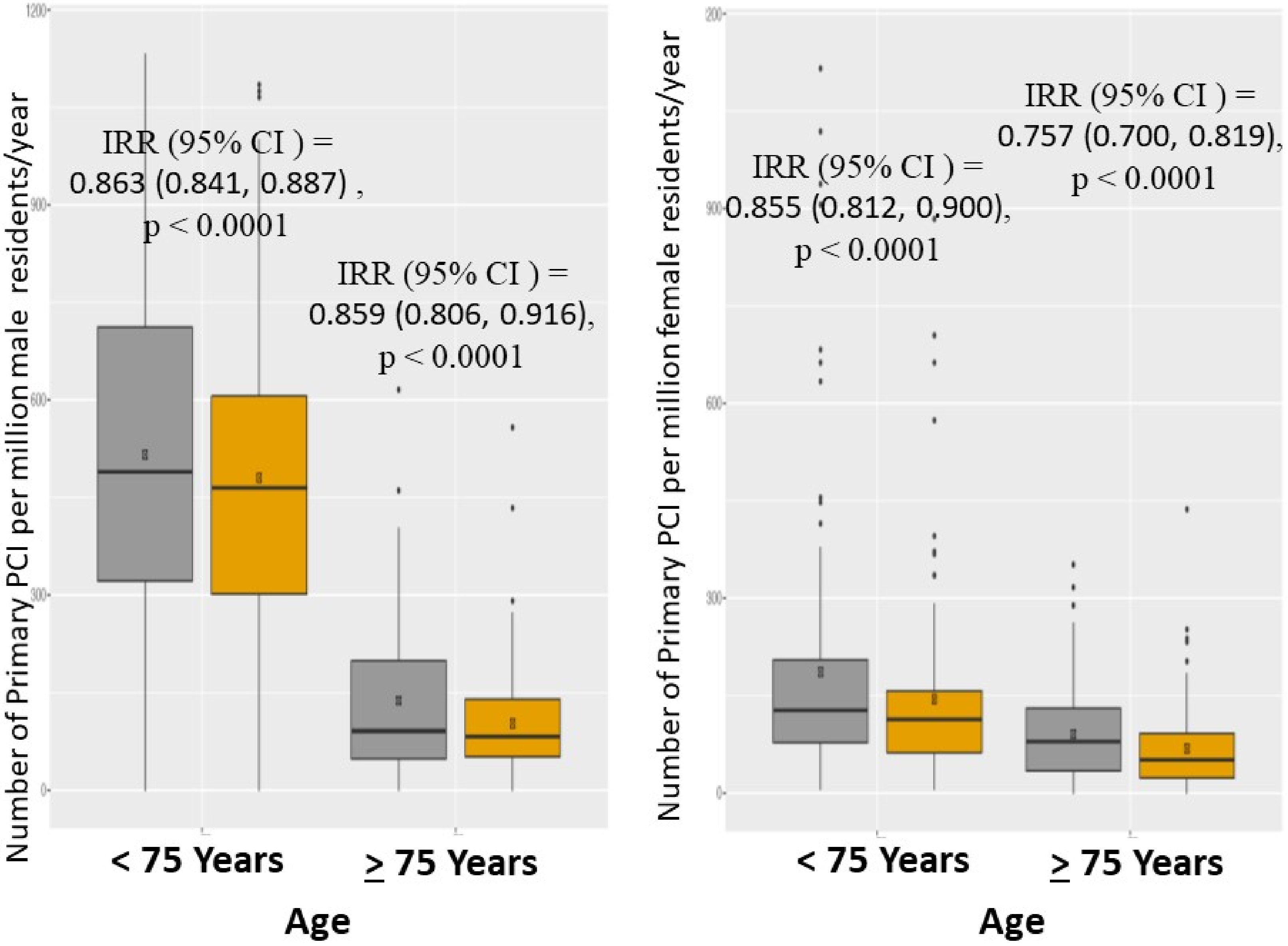

Number of Primary PCI per million male residents/year
IRR (95% CI) = 0.863 (0.841, 0.887) , p < 0.0001
IRR (95% CI) = 0.859 (0.806, 0.916), p < 0.0001
< 75 Years
> 75 Years
Age
Number of Primary PCI per million female residents/year
IRR (95% CI) = 0.855 (0.812, 0.900), p < 0.0001
IRR (95% CI) = 0.757 (0.700, 0.819), p < 0.0001
< 75 Years
> 75 Years
Age

Figure 12S

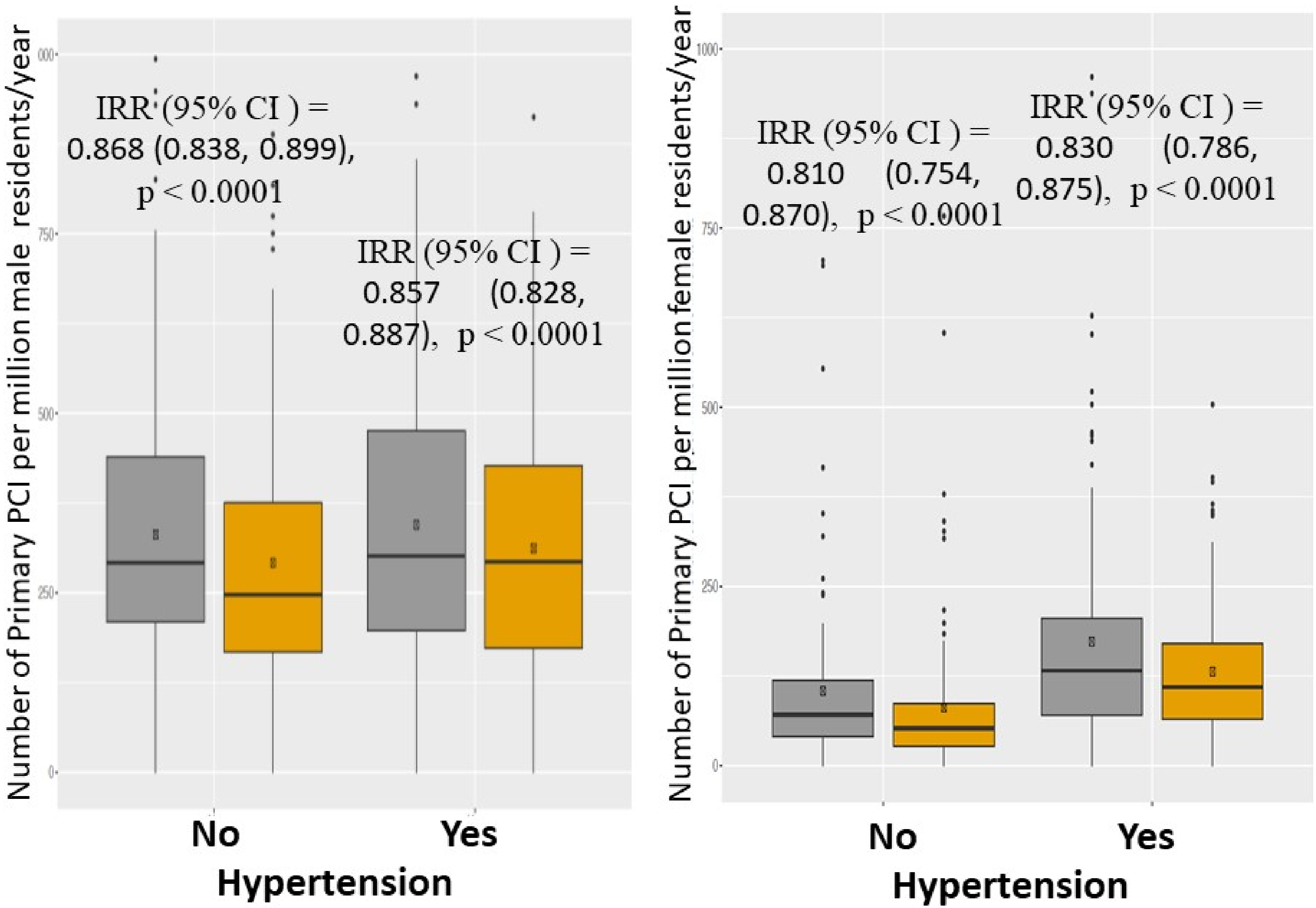
Number of Primary PCI per million male residents/year
IRR (95% CI) = 0.868 (0.838, 0.899), p < 0.0001
IRR (95% CI) = 0.857 (0.828, 0.887), p < 0.0001
750
500
250
0
No
Yes
Hypertension
Number of Primary PCI per million female residents/year
1000
750
500
250
0
IRR (95% CI) = 0.810 (0.754, 0.870), p < 0.0001
IRR (95% CI) = 0.830 (0.786, 0.875), p < 0.0001
No
Yes
Hypertension

Figure 13S

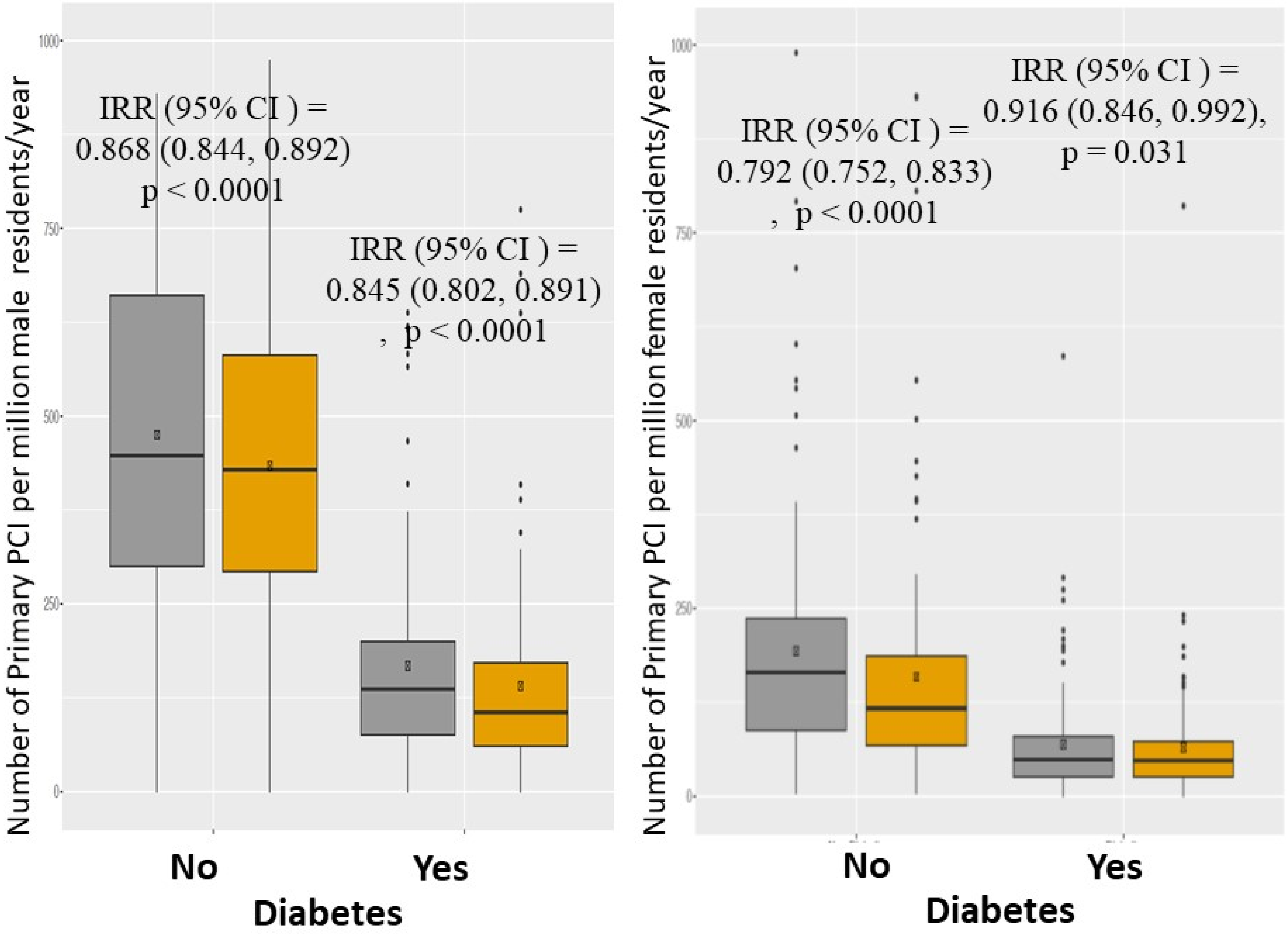

Number of Primary PCI per million male residents/year
IRR (95% CI) = 0.868 (0.844, 0.892) p < 0.0001
IRR (95% CI) = 0.845 (0.802, 0.891) , p < 0.0001
No
Yes
Diabetes
Number of Primary PCI per million female residents/year
IRR (95% CI) = 0.792 (0.752, 0.833) , p < 0.0001
IRR (95% CI) = 0.916 (0.846, 0.992), p = 0.031
No
Yes
Diabetes

Figure 14S

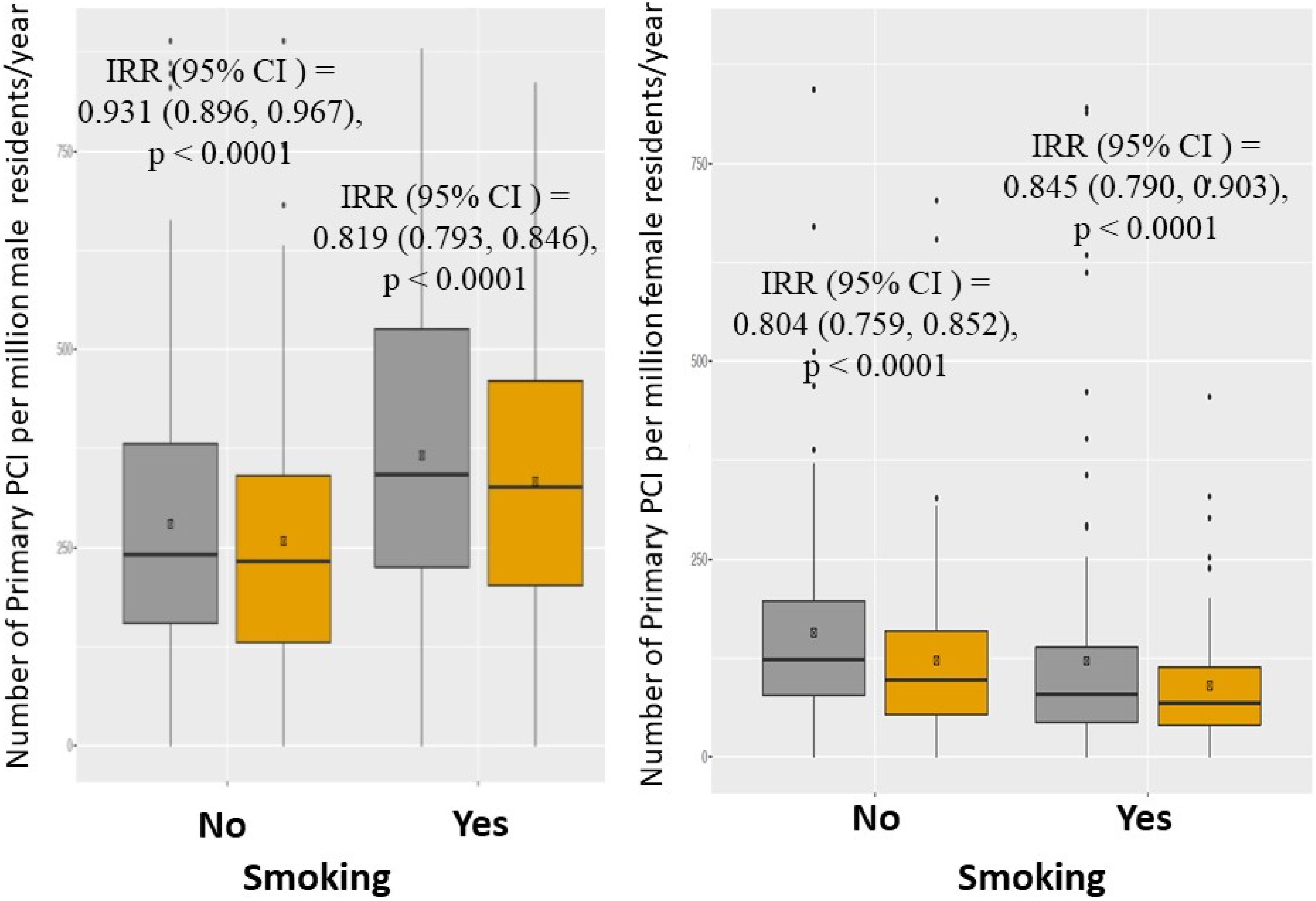

Number of Primary PCI per million male residents/year
IRR (95% CI) = 0.931 (0.896, 0.967), p < 0.0001
IRR (95% CI) = 0.819 (0.793, 0.846), p < 0.0001
750
500
250
0
No
Yes
Smoking
Number of Primary PCI per million female residents/year
IRR (95% CI) = 0.845 (0.790, 0.903), p < 0.0001
IRR (95% CI) = 0.804 (0.759, 0.852), p < 0.0001
750
500
250
0
No
Yes
Smoking

Figure 15S

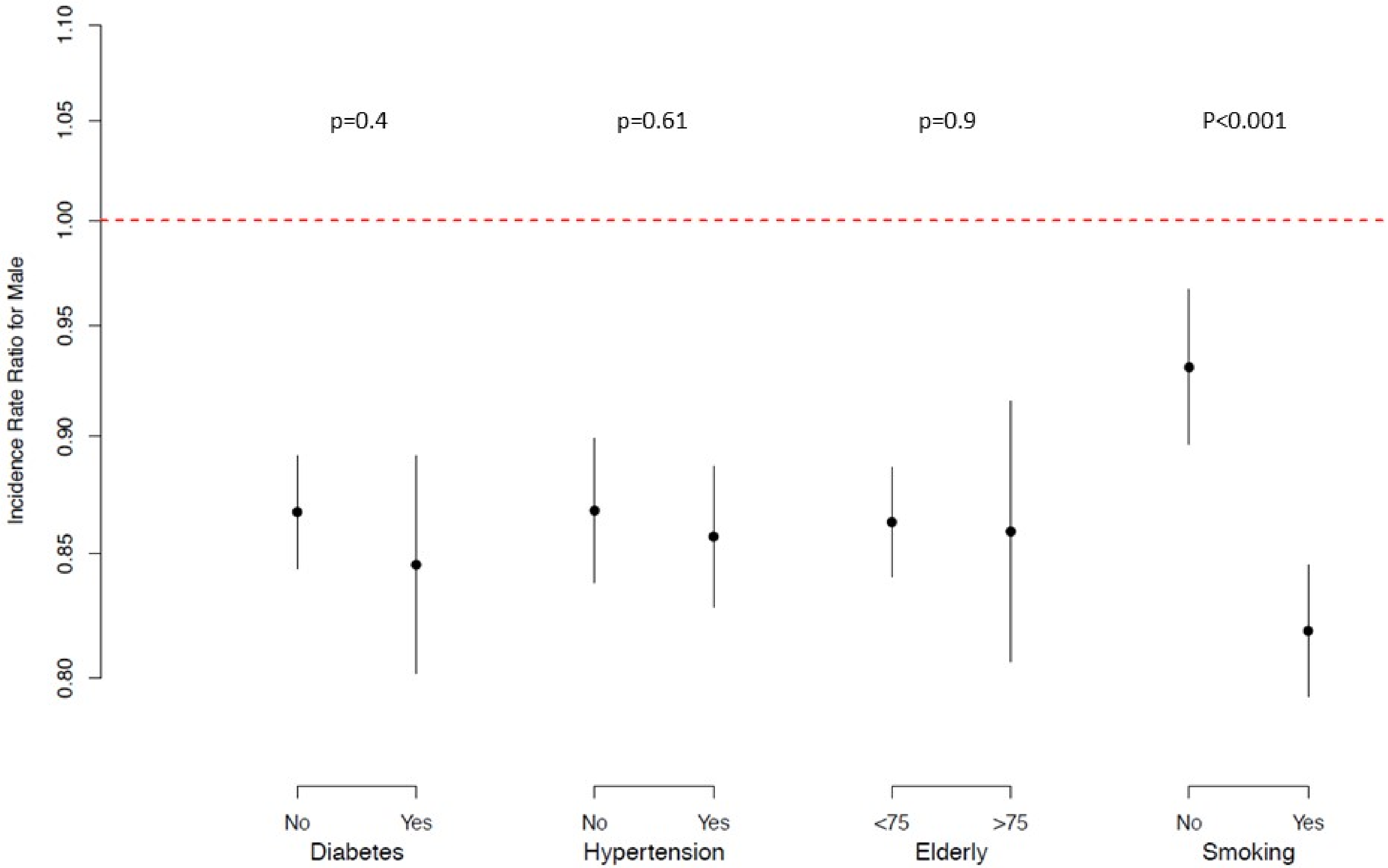

1.10
1.05
1.00
0.95
0.90
0.85
0.80
Incidence Rate Ratio for Male
p=0.4
p=0.61
p=0.9
P<0.001
No
Yes
Diabetes
No
Yes
Hypertension
<75
>75
Elderly
No
Yes
Smoking

Figure 16S

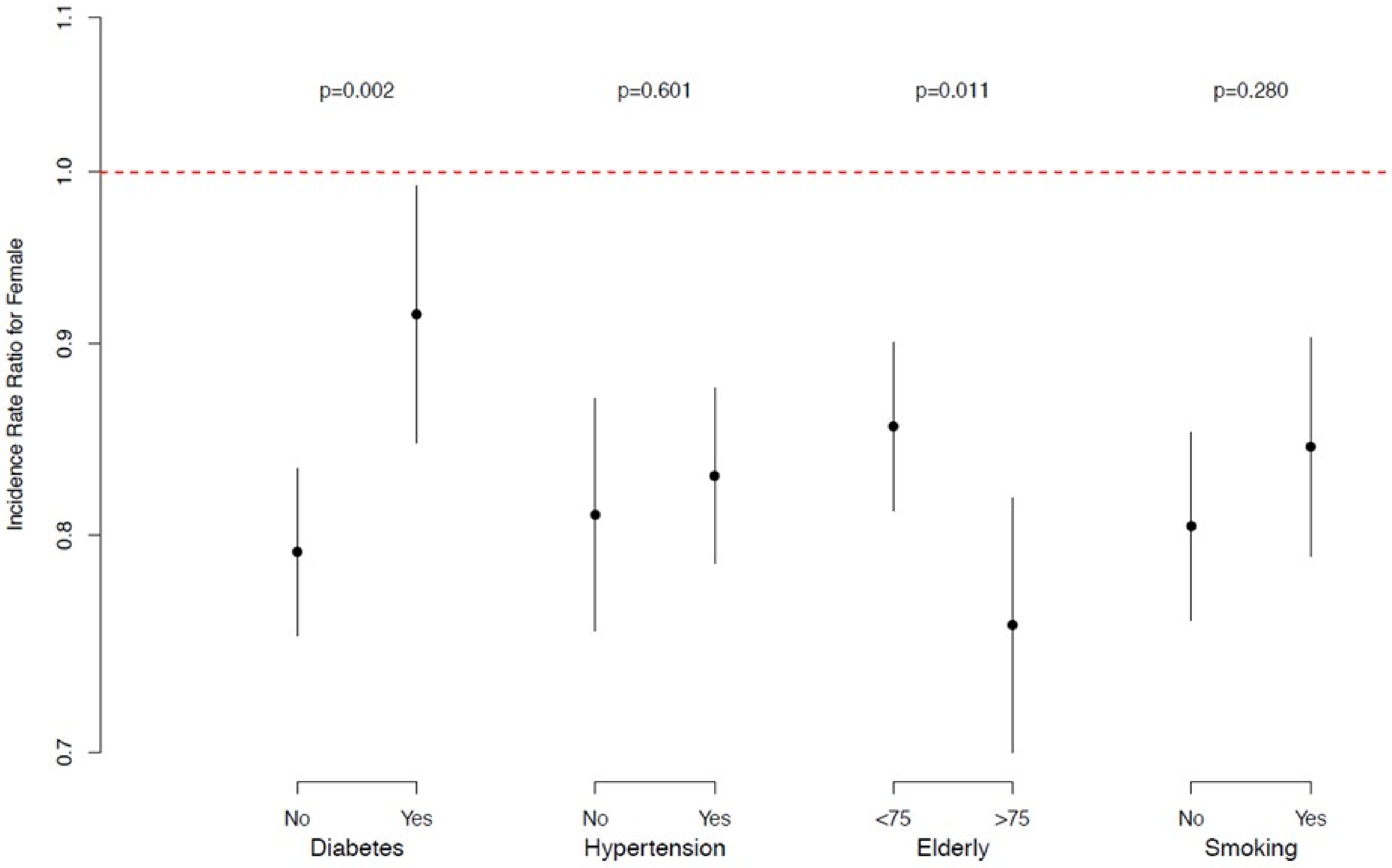

Incidence Rate Ratio for Female
1.1
1.0
0.9
0.8
0.7
p=0.002
p=0.601
p=0.011
p=0.280
No
Yes
Diabetes
No
Yes
Hypertension
<75
>75
Elderly
No
Yes
Smoking
